# Short-term effects of air pollution on hospitalization for acute lower respiratory infections in children: a time-series analysis study from Lanzhou, China

**DOI:** 10.1186/s12889-023-16533-7

**Published:** 2023-08-25

**Authors:** Wancheng Zhang, Jianglong Ling, Runping Zhang, Jiyuan Dong, Li Zhang, Rentong Chen, Ye Ruan

**Affiliations:** School of Public Health, Lanzhou University, Lanzhou, 730000 P. R. China

**Keywords:** Air pollution, Children, ALRI, Pneumonia, Bronchiolitis, Relative risk

## Abstract

**Background:**

Short-term exposure to air pollution is associated with acute lower respiratory infections (ALRI) in children. We investigated the relationship between hospitalization for ALRI in children and air pollutant concentrations from January 1, 2014 to December 31, 2020 in Lanzhou City.

**Methods:**

We collected data on air pollutant concentrations and children’s hospitalization data during the study period. A time series regression analysis was used to assess the short-term effects of air pollutants on ALRI in children, and subgroup analyses and sensitivity analyses were performed.

**Results:**

A total of 51,206 children with ALRI were studied, including 40,126 cases of pneumonia and 11,080 cases of bronchiolitis. The results of the study revealed that PM_2.5_, PM_10_, SO_2_ and NO_2_ were significantly associated with hospitalization for ALRI in children aged 0–14 years. For each 10 µg/m^3^ increase in air pollutant concentration in lag0-7, the relative risk of ALRI hospitalization in children due to PM_2.5_, PM_10_, SO_2_ and NO_2_ increased by 1.089 (95%CI:1.075, 1.103), 1.018 (95%CI:1.014, 1.021), 1.186 (95%CI:1.154. 1.219) and 1.149 (95%CI:1.130, 1.168), respectively.

**Conclusions:**

PM_2.5_, PM_10_, SO_2_ and NO_2_ short-term exposures were positively associated with ALRI, pneumonia and bronchiolitis hospitalizations in Lanzhou, China. Local governments should make efforts to improve urban ambient air quality conditions to reduce hospitalization rates for childhood respiratory diseases.

**Supplementary Information:**

The online version contains supplementary material available at 10.1186/s12889-023-16533-7.

## Introduction

Previous studies demonstrated that short-term exposure to air pollutants could cause various adverse health effects in people of different ages, such as respiratory and cardiovascular diseases [1–5]. Children have immature respiratory development, narrow bronchi, higher lung ventilation rates and lower immune capacity [6]. Therefore, children are more vulnerable to air pollution than adults. Acute lower respiratory infections (ALRI), including pneumonia and bronchiolitis, are a frequent respiratory disease and account for nearly one-fifth of the world’s mortality in young children and hospitalization for ALRI can account for 30–50% of pediatric hospitalizations [7, 8]. ALRI poses a huge threat to the life and health of children.

Currently, a number of studies have shown a strong correlation between air pollution and ALRI in children [9–14]. For instance, a Korean study found that exposure to fine particulate matter (PM_2.5_) was associated with ALRI hospitalization in children [15]. A study found an association between PM_2.5_ and hospitalization for pneumonia and bronchitis in children in the United States [16].

Similarly, the harmful effects of air pollutants on the respiratory tract of children have been found in several studies in China [17–19]. However, studies on the association of air pollutants with lower respiratory tract infections in children have focused on the harmful effects of ambient particulate matter in China and the majority of studies were from economically developed regions in eastern China. Only a few involved the effects of gaseous air pollutants on lower respiratory tract diseases in children [20–22]. Environmental particulate matter, such as PM_2.5_ and coarse particulate matter (PM_10_), can trigger oxidative stress and inflammation in lung tissues directly and can also enter the bloodstream through the respiratory tract. It causes not only damage to the respiratory tract but also extensive damage to the cardiovascular system [23]. Gaseous air pollutants, such as sulfur dioxide (SO_2_) and nitrogen dioxide (NO_2_), often damage mucous membranes and cause constriction of the airways [24]. Nonetheless, there is a limited amount of research on the correlation between air pollutants and hospitalization for ALRI in children.

The social and economic development level and the air pollution level will vary according to the geographical environment [25]. Lanzhou City is located in the inland area of northwest China, and its economic development is relatively backward. Air pollution is more serious than in southeastern China. Lanzhou is a typical river valley city, and the topographical features make air pollutants tend to disperse untimely. The adverse effects of air pollution are exacerbated by the gradual increase in hazy weather.

We performed this study to assess the association of major air pollutants (PM_2.5_, PM_10_, SO_2_, and NO_2_) with hospitalization for pediatric ALRI in Lanzhou, China. We also analyzed the association of air pollutants with hospitalization for specific etiologies, namely pneumonia and bronchitis. In addition, we also performed gender, age and seasonal stratified analyses.

## Methods

### Study area

Lanzhou is an inland city in northwest China. It is geographically situated on the Loess Plateau and has a temperate continental climate with relatively dry climate and low precipitation throughout the year. Lanzhou is an important transportation city in China and an important node city of the Silk Road Economic Belt. Lanzhou City has eight county-level administrative regions under its jurisdiction. Our study area is the four main urban areas (Chengguan, Qilihe, Xigu and Anning).

### Hospitalizations data

We collected information on inpatients aged 0–14 years from January 1, 2014 to December 31, 2020 from seven large general hospitals in Lanzhou City. These data included patient case number, admission diagnosis, age, gender, time of admission, and home address. Admission diagnoses were coded according to the International Classification of Diseases, 10th Revision (ICD-10). We selected the diseases involved in this study according to ICD-10 codes: acute lower respiratory infections (J12-J18 and J20-J22), pneumonia (J12-J18) and bronchiolitis (J20-J21) [26, 27]. We excluded patients whose home address was not within the study area for visits. We removed patients with errors and missing gender and age. To exclude double counting of the same patient due to multiple visits, we included only the first visit record of the patient during the study period. Because multiple visits by a patient may be due to referrals, transfers, or follow-up visits.

### Air pollution and meteorological data

**Fig. 1:**
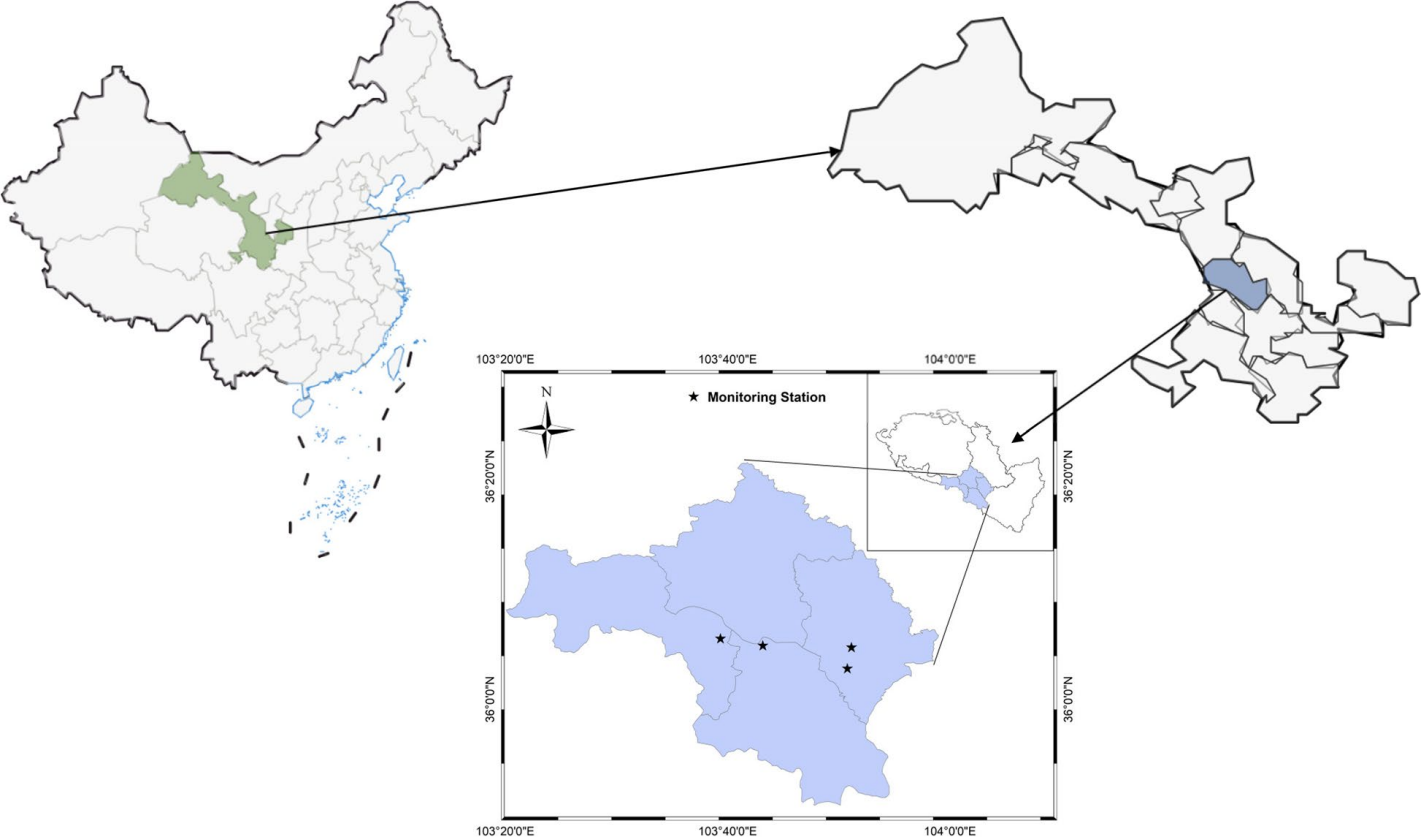
Location of air quality monitoring stations in Lanzhou

During the study period, air pollutant concentration data were recorded at four national air quality testing stations in urban Lanzhou. The arithmetic mean of the 24-h average concentration values of the major air pollutants at each monitoring station was taken. Four air pollutants that are closely related to respiratory diseases were selected: PM_2.5_, PM_10_, SO_2_, and NO_2_ [28]. In this study, as individual exposure levels could not be accurately estimated, the average of all pollutant monitoring station data was considered as the previously defined individual exposure level. Lanzhou City is a river-valley type city, and the terrain is characterized by a narrow east–west direction and a narrow north–south direction facing the mountains. Residential areas are mainly concentrated on both sides of the city roads, and the location of the air quality monitoring station is not more than 15 km away from the residential areas [29, 30]. Therefore, the monitoring data from the air quality testing station can well represent the exposure concentration of the residents. Figure 1 shows the location of the four national air quality monitoring stations in Lanzhou. We obtained meteorological data, including daily average temperature and average relative humidity data from China Meteorological website Data Service Center (http://data.cma.cn) for the periods from January 1, 2014 to January 31, 2020. During the study period, we did not find any missing pollutant concentration data and meteorological data.

### Statistical analysis

Time series studies have been widely utilized to study the short-term effects of air pollution on human health. Prior to modeling, we provided a descriptive statistical analysis of the relevant data. Similarly, we analyzed the correlations between major air pollutants and meteorological factors using Spearman correlation coefficients. Hospitalization of children for ALRI is considered a small probability event and follows a quasi-Poisson distribution. We used generalized linear models and distributional lag models with a family of quasi-Poisson distributions to investigate the effect of air pollutants on hospitalization for ALRI in children. We formulated a cross-basis for air pollutant with a linear function in both the predictor space and the lag structure stratification with constraints. Meteorological factors and long-term trends are controlled by natural cubic spline functions. By combining the previous relevant studies [31–33], we constructed the following statistical model:

$$\mathrm{Log}\left[E\left({Y}_{t}\right)\right]=\alpha +cb({x}_{t,l},\mathrm{df}=3)+ns\left(time,\mathrm{ df}=7\right)+ns\left({temp}_{t},\mathrm{ df}=3\right)+ ns\left({rh}_{t},\mathrm{ df}=3\right)+DOW+Holiday$$

where *t* is the observation day. *Y*_*t*_ is the dependent variable, indicating the number of children admitted for ALRI on day *t*. *E(Y*_*t*_*)* is the expected value of hospital admissions due to ALRI for children on that day. *α* is the intercept in the model. *cb* denotes the cross-basis function, which determines the matrix with the linear and lag matrices of air pollutants using a linear function and a natural cubic spline function with three dfs, respectively. *x*_*t*_ indicates PM_2.5_, PM_10_, SO_2_ and NO_2_. *l* is the lag of the *x*_*t*_. *ns* is the natural cubic spline function, *time* is the time variable, *tempt* is the average temperature on day *t*, *rh*_*t*_ is the average relative humidity on day *t*, and *df* is the degree of freedom. *DOW* is a dummy variable denoting the control week effect, and *Holiday* is a dummy variable denoting the control public holiday. We set the degrees of freedom for the variables of interest in this study based on previous related studies [28, 34]. The ultimate incorporated model the degrees of freedom for time, temperature, and humidity were 7, 3, and 3 respectively.

Based on the experience of previous related studies [35], we constructed eight single-lag structures (lag0-lag7) and seven cumulative lag structures (lag0-1 ~ lag0-7) to estimate the short-term effects of air pollutants on children’s disease (ALRI, pneumonia and bronchiolitis) hospitalization. The estimates were expressed as relative risk (RR) values and corresponding 95% confidence intervals (CIs) for each 10 µg/m^3^ increase in concentrations of PM_2.5_, PM_10_, SO_2_, and NO_2_ for childhood ALRI hospitalizations. We further conducted subgroup analyses to estimate the effect of air pollutant concentration variations on age (< 5 years and 5–14 years) [26, 27], gender (boy or girl) and season (cool season from October to March and warm season from April to September). We then further assessed the significant differences between the effect estimates of the stratified analyses by calculating 95% CIs using the following equation:

$${\widehat{(\mathrm{Q}}}_{1}-{\widehat{\mathrm{Q}}}_{2})\pm 1.96\sqrt{{\mathrm{S}{\widehat{\mathrm{E}}}_{1}}^{2}+{\mathrm{S}{\widehat{\mathrm{E}}}_{2}}^{2}}$$

where ${\widehat{\mathrm{Q}}}_{1}$ and ${\widehat{\mathrm{Q}}}_{2}$ indicate the estimates for two subgroups (e.g., boy and girl), $\mathrm{S}{\widehat{\mathrm{E}}}_{1}$ and $\mathrm{S}{\widehat{\mathrm{E}}}_{2}$ are their appropriate standard errors.

We selected the maximum effect time for the short-term effects of air pollutants on children’s ALRI for sensitivity analysis. Based on previous related studies, we assessed the stability of the model results by constructing a two-pollutant model and varying the degrees of freedom of the time variable (df = 6 ~ 10) [36]. All statistical analyses for this study were performed using R software. Two-sided tests with P values less than 0.05 were considered statistically significant.

## Results

### Descriptive statistics of children’s ALRI hospitalization, air pollutants and meteorological factors

From January 1, 2014 to December 31, 2020, a total of 51,206 ALRI patients 0–14 years old were hospitalized in Lanzhou City, of which 40,126 (78.36%) were admitted for pneumonia and 11,080 (21.64%) were admitted for bronchiolitis. Table 1 summarizes the descriptive statistics of daily hospitalizations, air pollutant concentrations and meteorological factors. During the study period, the average daily ALRI hospitalization was 20 (range 0–91), of which 16 were pneumonia-related (range 0–81) and 4 were bronchiolitis-related (range 0–18). The mean concentrations of PM_2.5_, PM_10_, SO_2_ and NO_2_ during the study period were 47.8, 112.0, 20.6 and 48.2 µg/m^3^, respectively. The average daily temperature was 11.1 °C and the average relative humidity was 51.6%.

**Table 1:** Descriptive statistics for daily hospitalizations, air pollutants and meteorological factors in Lanzhou, China, 2014–2020

	**Mean**	**SD**	**Percentile**				
			**Minimum**	**25th**	**50th**	**75th**	**Maximum**
**Daily hospitalizations (n)**							
ALRI	20	15	0	10	16	26	91
Pneumonia	16	13	0	7	12	21	81
Bronchiolitis	4	3	0	2	4	6	18
**Air pollutions (µg/m**^**3**^**)**							
PM_2.5_	47.8	26.1	8.9	30.7	41.8	57.8	275.1
PM_10_	112.0	79.8	16.8	69.6	97.4	133.9	1484.5
SO_2_	20.6	13.5	3.5	10.3	16.6	27.2	80.5
NO_2_	48.2	17.6	10.1	36.4	46.2	55.7	151.1
**Meteorological variables**							
Temperature, ℃	11.1	9.9	-12.3	2.26	12.2	19.8	30.4
Relative humidity, %	51.6	15.1	11.7	40.1	52.0	62.6	96.1

**Fig. 2:**
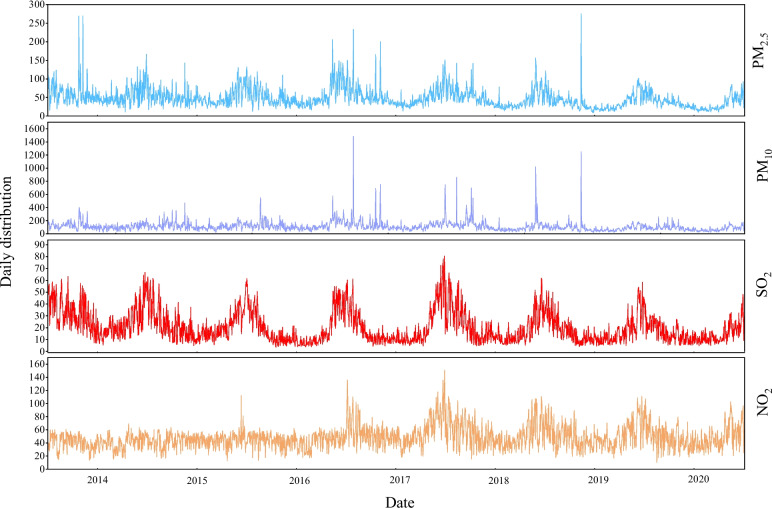
Temporal changes in air pollutant concentrations in 2014–2020

Figure 2 represents the temporal trends of the main air pollutants during the period of study and what we can know is that air pollution has seasonal trends, especially PM_2.5_ and SO_2_.

**Fig. 3:**
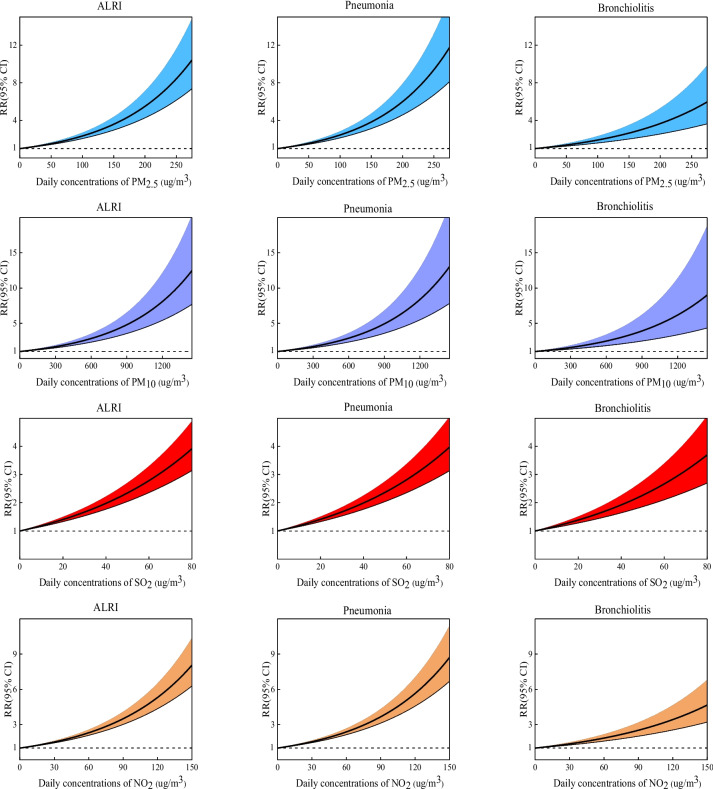
The exposure-response curves between air pollutants and ALRI hospitalizations in children

Figure 3 shows the concentration–response relationships of four air pollutants with ALRI, pneumonia, and bronchitis. We can find that increased concentrations of PM_2.5_, PM_10_, SO_2_ and NO_2_ all lead to an increased relative risk of hospitalization for ALRI, pneumonia and bronchiolitis, and none of them has a threshold.

Table S1 shows the Spearman correlation coefficients of the main air pollutants and meteorological factors. All of the air pollution are positively correlated with each other. Among them, PM_2.5_ is highly positively correlated with PM_10_ with a correlation coefficient of 0.85. Air pollutants are negatively correlated with both temperature and relative humidity.

**Fig. 4:**
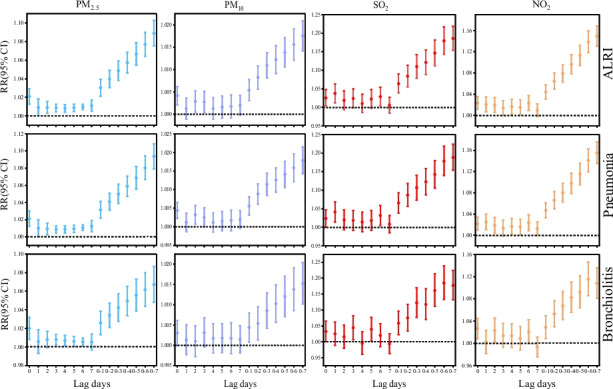
Relative risk (95%CI) of overall and cause-specific ALRI hospital admissions associated with an increase of 10 µg/m^3^ in PM_2.5_, PM_10_, SO_2_ and NO_2_ along different lag structures, using single pollutant models

Figure 4 and Table S2 show the short-term effects of the four air pollutants on children hospitalized (ALRI, pneumonia and bronchiolitis) on the day of admission and at the various lag days.

PM_2.5_ had a harmful effect on ALRI and pneumonia at different lag days and both had a maximum lag effect at lag0-7, with RR values of 1.089 (95% CI:1.075,1.103) and 1.094 (95% CI:1.079, 1.109), respectively. PM_2.5_ had a harmful effect on bronchiolitis at most lag days, except for lag1, lag2 and lag7, similarly the maximum lag effect was seen in lag0-7, RR = 1.067, (95% CI:1.048,1.086).

PM_10_ had a similar effect on ALRI and pneumonia admissions. In the single lag structure, there was a harmful effect only at lag0, lag2, and lag3, with no harmful effect at any other single lag days. However, there was a harmful effect at all cumulative lag days, and the largest effect occurred at lag0-7, RR = 1.018 (95% CI:1.014,1.021) and RR = 1,018 (95% CI:1.043,1.021), respectively. However, the effect of PM10 on bronchiolitis was harmful only at the cumulative lag days with a maximum effect RR = 1.015 (95% CI:1.010, 1.020) at lag0-7.

SO_2_ has a similar effect on ALRI and pneumonia. The effect was harmful only at lag0, lag1 and lag6 in single lag days. However, it was harmful at all cumulative lags, and the maximum lag effect occurred at lag0-7, RR = 1.186 (95% CI:1.154,1.219) and RR = 1,188 (95% CI:1.154,1.224), respectively. The effect of SO_2_ on bronchiolitis at lag0, lag3, lag5 and lag0-1 ~ lag0-7 was harmful, with a maximum effect of RR = 1.185 (95% CI:1.133, 1.239) at lag0-6.

NO_2_ had a comparable effect on ALRI and pneumonia admissions. At all single lag days, the effect estimates of the effects were not harmful only at lag3 and lag7. However, at all cumulative lag days, NO_2_ had a harmful effect on ALRI and pneumonia admissions, with the largest lag effects occurring at lag0-7, with RRs of 1.149 (95% CI:1.130,1.168) and 1.155 (95% CI:1.135,1.176), respectively. The effect is harmful for NO_2_ on bronchiolitis at lag0, lag2, lag6 and lag0-1 ~ lag0-7 with maximum effect RR = 1.115 (95% CI:1.085, 1.146) at lag0-6.

### Subgroup analysis by gender, age and season

Table 2 shows the relative risk estimates for different air pollutants for each disease. There were no statistically significant between-group differences for different diseases stratified by gender for different air pollutant exposures (*p* > 0.05), but the pollutant exposures all showed deleterious effects (Table S3). Age subgroup results showed statistically significant differences in the harmful effects of only PM_2.5_ and NO_2_ on hospitalization for both ALRI and pneumonia (*p* < 0.05). The harmful effects of PM_2.5_ are stronger in the < 5 years age group, while those of NO_2_ are stronger in the 5–14 years age group. No results with significant differences for age stratification were observed for any of the remaining pollutant exposures (*p* > 0.05), but the pollutant exposures all had harmful effects (Table S4). Seasonal subgroup results showed statistically significant differences in the harmful effects of PM_2.5_ on hospitalizations for ALRI and bronchiolitis, and PM_10_ and NO_2_ on hospitalizations for ALRI, pneumonia and bronchiolitis (*p* < 0.05). The effects of short-term exposure to the four air pollutants on childhood ALRI hospitalization were stronger in the cold season compared to the warm season. However, pollutant exposure had deleterious effects on ALRI and pneumonia hospitalization in both warm and cold seasons. In contrast, the harmful effects of pollutant exposure on bronchiolitis hospitalization were only observed in the cold season (Table S5).

**Table 2:** Relative risk (95%CI) in daily overall and cause-specific ALRI hospital admissions per 10 µg/m^3^ increment in PM_2.5_, PM_10_, SO_2_ and NO_2_, stratified by gender, age and season

Disease	Air pollution	Overall	Gender		Age		Season	
			Male	Female	< 5y	5-14y	Warm	Cold
ALRI	PM_2.5_^**a**^	1.089 (1.075,1.103)	1.088 (1.074,1.103)	1.090 (1.073,1.106)	**1.095 (1.081,1.109)**	**1.067 (1.046,1.089)**	**1.041 (1.017,1.066)**	**1.082 (1.065,1.099)**
	PM_10_^**a**^	1.018 (1.014,1.021)	1.017 (1.014,1.021)	1.018 (1.014,1.022)	1.019 (1.015,1.022)	1.013 (1.008,1.019)	**1.009 (1.004,1.015)**	**1.019 (1.015,1.024)**
	SO_2_^**a**^	1.186 (1.154,1.219)	1.188 (1.153,1.224)	1.184 (1.145,1.224)	1.176 (1.142,1.210)	1.225 (1.174,1.277)	1.214 (1.128,1.305)	1.230 (1.189,1.273)
	NO_2_^**a**^	1.149 (1.130,1.168)	1.144 (1.124,1.165)	1.156 (1.133,1.179)	**1.139 (1.119,1.159)**	**1.186 (1.156,1.217)**	**1.065 (1.025,1.106)**	**1.169 (1.145,1.194)**
Pneumonia	PM_2.5_^**a**^	1.094 (1.079,1.109)	1.095 (1.097,1.111)	1.092 (1.074,1.110)	**1.100 (1.085,1.116)**	**1.071 (1.048,1.094)**	1.052 (1.025,1.079)	1.083 (1.065,1.101)
	PM_10_^**a**^	1.018 (1.014,1.021)	1.018 (1.014,1.022)	1.017 (1.013,1.022)	1.019 (1.016,1.023)	1.013 (1.007,1.019)	**1.010 (1.004,1.016)**	**1.019 (1.014,1.024)**
	SO_2_^**a**^	1.188 (1.154,1.224)	1.192 (1.154,1.231)	1.184 (1.142,1.227)	1.175 (1.139,1.212)	1.241 (1.186,1.299)	1.246 (1.148,1.353)	1.236 (1.192,1.282)
	NO_2_^**a**^	1.155 (1.135,1.176)	1.153 (1.130,1.175)	1.159 (1.134,1.184)	**1.146 (1.125,1.167)**	**1.192 (1.159,1.226)**	**1.072 (1.028,1.118)**	**1.171 (1.144,1.197)**
Bronchiolitis	PM_2.5_^**a**^	1.067 (1.048,1.086)	1.061 (1.039,1.084)	1.075 (1.049,1.103)	1.072 (1.051,1.093)	1.052 (1.016,1.090)	**1.005 (0.966,1.045)**	**1.076 (1.052,1.102)**
	PM_10_^**a**^	1.015 (1.010,1.020)	1.013 (1.007,1.019)	1.019 (1.012,1.026)	1.016 (1.010,1.021)	1.013 (1.002,1.023)	**1.006 (0.997,1.016)**	**1.019 (1.012,1.026)**
	SO_2_^**b**^	1.185 (1.133,1.239)	1.184 (1.122,1.249)	1.186 (1.113,1.264)	1.193 (1.135,1.253)	1.166 (1.072,1.268)	1.088 (0.965,1.227)	1.243 (1.179,1.311)
	NO_2_^**b**^	1.115 (1.085,1.146)	1.116 (1.080,1.152)	1.116 (1.073,1.159)	1.109 (1.076,1.143)	1.135 (1.078,1.195)	**1.010 (0.946,1.078)**	**1.172 (1.134,1.212)**

In bold: statistically significant differences between groups

^a^The maximum effect of air pollutants on each disease was lag0-7 for the maximum lag day

^b^The maximum effect of air pollutants on each disease was lag0-6 for the maximum lag day

### Sensitivity analysis

Firstly, we chose the time of maximum effect of each air pollutant to verify the stability of the model by constructing a two-pollutant model. PM_2.5_ and PM_10_ are highly correlated with a Spearman correlation coefficient of 0.85, therefore we do not introduce them into the same model at the same time. Table S6 summarizes the results of the two-pollutant model. We can find that the effect between particulate matter and ALRI, pneumonia and bronchiolitis hospitalization is diminished after controlling for gaseous pollutants, but it is still statistically significant. Similarly, when we control for particulate matter, the effect between gaseous pollutants and their outcome changes ALRI, pneumonia and bronchiolitis hospitalizations is attenuated, but still statistically significant.

Secondly, we also chose the time of maximum effect of each air pollutant and performed sensitivity analysis by changing the degrees of freedom of the time variable (Table S7). We found that changing the degrees of freedom of time to 6, 8, 9 and 10 did not significantly change the short-term effects of air pollutants on ALRI, pneumonia and bronchiolitis.

## Discussion

This study assessed the correlation between short-term exposure to air pollution and the risk of hospitalization for ALRI in children from 2014 to 2020 in Lanzhou, China, using a time-series analysis. Our findings suggest that short-term exposure to air pollutants leads to an increased risk of hospitalization for ALRI, pneumonia, and bronchitis in children after controlling for temperature and relative humidity. PM_2.5_ had a stronger deleterious effect compared to PM_10_. The estimated relative risk of childhood ALRI hospitalization from SO_2_ exposure was greater than that from NO_2_ at lag0-7. However, NO_2_ exhibited a deleterious effect on childhood ALRI hospitalization at all lag times, except at Lag7. In conclusion, our findings add to the evidence that air pollutants increase the risk of ALRI in children in developing countries and regions.

The harmful effects of particulate matter on children with ALRI are consistent with the findings of previous studies. Our study found that for every 10 µg/m^3^ increase in PM_2.5_ concentration, the relative risk of hospitalization for ALRI, pneumonia and bronchitis in children increased by 1.089 (95% CI: 1.075, 1.103), 1.094 (95% CI: 1.079, 1.1094) and 1.067 (95% CI: 1.048, 1.086) respectively at lag0-7. For each 10 µg/m^3^ increase in PM_10_ concentration, the relative risk of hospitalization for ALRI and pneumonia in children increased by the same amount, RR = 1.018 (95% CI:1.014,1.021) at lag0-7. The relative risk of hospitalization for bronchitis was increased by 1.015 (95% CI:1.01,1.02) at lag0-7. The harmful effect has been found in several other studies. For example, a study in Vietnam found that every 10 µg/m^3^ increase in PM_2.5_ concentration was associated with a 3.51 (95% CI: 0.96,6.12) increase in the risk of hospital admission for ALRI in children at lag3 [11]. A Korean study also found a strong linear correlation between PM_2.5_ and childhood ALRI hospitalization and an increase of 1.20% (95% CI: 0.71, 1.71) in ALRI hospitalization for every 10 µg/m^3^ increase in PM_2.5_ concentration at lag0-7 [15]. A study conducted in Sichuan, China, found a 1.23% (95% CI: 0.21,2.26) increase in the risk of hospitalization for childhood bronchitis for every 10 µg/m^3^ increase in PM_2.5_ at lag0-7 [28]. Another study in the region, Sichuan, found that for each 10 µg/m^3^ increase in PM_2.5_ at lag4 and PM_10_ at lag0-10, the relative risk of hospitalization for childhood pneumonia was 1.0064 (95% CI:1.0004,1.0124) and 1.0168 (95% CI:1.0089,1.0248), respectively [19]. A study in Jinan, China found a correlation between PM_2.5_ and PM_10_ and childhood pneumonia admissions, with each 10 µg/m^3^ increase in PM_2.5_ and PM_10_ associated with 6% (95% CI:1.02,1.10) increase in lag1 and 4% (95% CI:1.00,1.08) increase in lag2, respectively [37]. A study in Hefei, China, showed that for every 10 µg/m^3^ increase in PM_2.5_ and PM_10_ concentrations at lag0-2, the risk of hospitalization for pneumonia increased by 1.21% (95% CI:0.34%, 2.09%) and 1.10% (95% CI:0.44%, 1.76%), respectively [38]. These studies also showed a stronger effect of PM_2.5_ compared to PM_10_ on children’s ALRI hospitalization. Unlike our study, the time to maximum harmful effects did not occur at the same time for different particulate matter and their risk values for hospitalization for ALRI in children were not identical. It could be caused by various regions, particulate matter composition in the urban environment and social lifestyles and habits.

We found that PM_2.5_ had the strongest harmful effect on hospitalization for pneumonia compared to fine bronchitis and ALRI at lag0-7, RR = 1.094 (95% CI:1.079, 1.109). Components of PM_2.5_, such as elemental carbon, organic carbon and nitrate, are associated with hospitalization for respiratory diseases in children such as pneumonia and bronchitis, and are most correlated with pneumonia [39]. Due to the complex composition and smaller aerodynamic diameter of PM_2.5_, it can easily enter deeper into the respiratory tract and deposit in the lung tissue. It can enter the circulatory system through the blood gas barrier and cause more extensive damage to the body. PM_10_ has a relatively small impact on children’s ALRI hospitalization, but still has harmful effects. Currently, the toxicological mechanisms of PM_2.5_ damage to the respiratory system are mainly free radical peroxidation, imbalance of intracellular calcium homeostasis and inflammatory damage [40].

Gaseous air pollutants also have harmful effects on children with ALRI. A Brazilian study found a 7% relative risk of pneumonia hospitalization for NO_2_ at lag1 [41]. A study conducted in eastern China similarly found that for each 10-unit increase in SO_2_ and NO_2_, pediatric pneumonia hospitalizations increased by 11.21% (95% CI:4.70%, 18.10%) and 5.42% (95% CI:3.07%, 7.82%), respectively [35]. SO_2_ is mainly originated from the combustion of coal, oil and other sulfur-containing minerals. When SO_2_ enters the respiratory tract, it can form the corresponding derivatives, the sodium bisulfite and sulfite, which are toxic to the respiratory system [42]. NO_2_ is primarily sourced from vehicle exhaust. Short-term exposure to NO_2_ may enhance airway inflammation by modulating Th1/Th2 differentiation and activating the JAK-STAT pathway, thereby causing lung injury [43].

The results of gender stratified analysis showed that PM_2.5_, PM_10_ and SO_2_, except NO_2_, had a stronger harmful effect on ALRI hospitalization in boys. A Korean study similarly found a stronger association between PM_2.5_ exposure and ALRI in boys (1.31%, 95% CI: 0.75, 1.86) [15]. It is consistent with several studies in other regions of China, such as Hefei and Guangzhou [17, 38]. However, in contrast to these studies, we found that NO_2_ had a stronger harmful effect on girls. This might be attributed to the more severe air pollution conditions in Lanzhou compared to the eastern regions of China. During our study period, the average daily NO_2_ concentration in Lanzhou was 48.2 µg/m^3^. In comparison, the average NO_2_ concentrations in Ningbo and Guangzhou, China were 39.7 µg/m^3^ and 45.2 µg/m^3^ [17, 35]. Gender differences in the effects of air pollution on respiratory disease in children still exist and the corresponding mechanisms are unclear. The different sensitivity of boys and girls to air pollutants may be due to the interaction of anatomical, genetic and physiological differences as well as behavioral factors [44]. Furthermore, boys spend more time outdoors than girls, which may also contribute to boys’ greater vulnerability to air pollution.

Age-stratified results showed that PM_2.5_, PM_10_ and SO_2_ were more significant hazards for children hospitalized with ALRI in the < 5 years age group. The results are compatible with previous studies [38]. Children in the < 5 years old age group are more susceptible to the effects of air pollution because of their weaker immune defenses and immature respiratory systems. NO_2_ was more associated with ALRI hospitalization in children aged 5–14 years. A study from southern Brazil also found NO_2_ to be associated with respiratory hospitalization only in children aged 6–15 years (IRR = 1.14, 95% CI:1.11,1.17) [45]. The main source of NO_2_ is traffic pollution. School-age children are more likely to spend more time outdoors and have more exposure to traffic pollution, so they are more affected by NO_2_.

Seasonal stratification analysis showed that the effects of PM_2.5_, PM_10_, SO_2_ and NO_2_ on children’s ALRI hospitalization were more powerful in the cold season. Cold stimulation impairs the ciliary function of the respiratory mucosa thereby inhibiting the clearance of pollutants [46]. Cold air increases the number of inflammatory cells in the lower respiratory tract, such as centrophages and macrophages [47]. In addition, the cold has a relationship with air pollution, such as the cooling effect of nitrate particles and organic carbon of PM_2.5_ components on the climate, and the possible coexistence of extreme cold and particulate pollution [48]. In addition, cold air and gaseous air pollutants have a combined damaging effect on the respiratory tract [49]. This all makes the impact of air pollutants on respiratory diseases more significant in the cold season.

The strengths of our study are as follows: to our knowledge, this is the first study in Lanzhou on the relationship between exposure to major air pollutants and hospital admissions for ALRI in children. We stratified the outcomes of childhood ALRI hospitalizations by pollutants, diseases (pneumonia and bronchitis), gender, age and season. There are some limitations in our study. Firstly, this is an ecological study and, similar to other studies, we used averages from fixed national air quality monitoring stations as an indicator of individual exposure levels, which inevitably leads to exposure measurement error. Secondly, our study did not include individual confounding factors such as second-hand smoke exposure, personal history of disease, activity patterns, etc., which may also have an impact on individual exposure.

## Conclusion

Our findings show that PM_2.5_, PM_10_, SO_2_ and NO_2_ short-term exposures were positively associated with ALRI, pneumonia and bronchiolitis hospitalizations in Lanzhou, China. Moreover, PM_2.5_, PM_10_ and SO_2_ had stronger effects on the boy group and the 5–14 years age group, while NO_2_ had the opposite but still significant deleterious effects. All pollutants were stronger for children ALRI hospitalization in the cold season. Therefore, efforts should be made to improve urban ambient air quality conditions to reduce hospitalization rates for respiratory diseases in children.

### Supplementary Information

**Additional file 1: Table S1.** Spearman correlation between air pollutions and meteorological factors in Lanzhou, China, 2014–2020. **Table S2.** Relative risk (95% CI) of single-pollutant model results in hospital admissions with ALRI, pneumonia and bronchiolitis associated with a 10 µg/m^3^ increase in air pollutant concentrations with different lag days. **Table S3.** Relative risk (95% CI) of single-pollutant model results in hospital admissions with ALRI, pneumonia and bronchiolitis associated with a 10 µg/m^3^ increase in air pollutant concentrations with different lag days by gender. **Table S4.** Relative risk (95% CI) of single-pollutant model results in hospital admissions with ALRI, pneumonia and bronchiolitis associated with a 10 µg/m^3^ increase in air pollutant concentrations with different lag days by age. **Table S5.** Relative risk (95% CI) of single-pollutant model results in hospital admissions with ALRI, pneumonia and bronchiolitis associated with a 10 µg/m^3^ increase in air pollutant concentrations with different lag days by season. **Table S6.** Relative risk (95% CI) of ALRI, pneumonia and bronchiolitis hospitalizations associated with a 10 µg/m^3^ increase in air pollutant concentrations in single and two-pollutant models. **Table S7.** Relative risk (95% CI) in hospital admissions for ALRI, pneumonia and bronchiolitis associated with a 10μg/m^3^ increase in air pollutant concentrations in sensitivity analyses.

## Data Availability

The datasets generated and analyzed during the current study are not publicly available due the data is collected for administrative purposes but are available from the corresponding author on reasonable request.
