# Supplementary material for: Short-term effects of air pollution on hospitalization for acute lower respiratory infections in children: a time-series analysis study from Lanzhou, China
Source: BMC Public Health. 2023 Aug 25;23:1629. doi: 10.1186/s12889-023-16533-7 (PMC10463321; doi:10.1186/s12889-023-16533-7)
Supplement: Supplementary file 1 — Additional file 1: Table S1. Spearman correlation between air pollutions and meteorological factors in Lanzhou, China, 2014–2020. Table S2. Relative risk (95% CI) of single-pollutant model results in hospital admissions with ALRI, pneumonia and bronchiolitis associated with a 10 µg/m3 increase in air pollutant concentrations with different lag days. Table S3. Relative risk (95% CI) of single-pollutant model results in hospital admissions with ALRI, pneumonia and bronchiolitis associated with a 10 µg/m3 increase in air pollutant concentrations with different lag days by gender. Table S4. Relative risk (95% CI) of single-pollutant model results in hospital admissions with ALRI, pneumonia and bronchiolitis associated with a 10 µg/m3 increase in air pollutant concentrations with different lag days by age. Table S5. Relative risk (95% CI) of single-pollutant model results in hospital admissions with ALRI, pneumonia and bronchiolitis associated with a 10 µg/m3 increase in air pollutant concentrations with different lag days by season. Table S6. Relative risk (95% CI) of ALRI, pneumonia and bronchiolitis hospitalizations associated with a 10 µg/m3 increase in air pollutant concentrations in single and two-pollutant models. Table S7. Relative risk (95% CI) in hospital admissions for ALRI, pneumonia and bronchiolitis associated with a 10μg/m3 increase in air pollutant concentrations in sensitivity analyses. [file 12889_2023_16533_MOESM1_ESM.docx]

**Supplemental Materials**

**Short-term effects of air pollution on hospitalization for acute lower respiratory infections in children: a time-series analysis study from Lanzhou, China**

Wancheng Zhang, Jianglong Ling, Runping Zhang, Jiyuan Dong, Li Zhang, Rentong Chen, Ye Ruan

School of Public Health, Lanzhou University, Lanzhou730000, PR China

**Table of Contents**

Table S1 Spearman correlation between air pollutions and meteorological factors in Lanzhou, China, 2014–2020.

Table S2 Relative risk (95% CI) of single-pollutant model results in hospital admissions with ALRI, pneumonia and bronchiolitis associated with a 10 µg/m^3^ increase in air pollutant concentrations with different lag days.

Table S3 Relative risk (95% CI) of single-pollutant model results in hospital admissions with ALRI, pneumonia and bronchiolitis associated with a 10 µg/m^3^ increase in air pollutant concentrations with different lag days by gender.

Table S4 Relative risk (95% CI) of single-pollutant model results in hospital admissions with ALRI, pneumonia and bronchiolitis associated with a 10 µg/m^3^ increase in air pollutant concentrations with different lag days by age.

Table S5 Relative risk (95% CI) of single-pollutant model results in hospital admissions with ALRI, pneumonia and bronchiolitis associated with a 10 µg/m^3^ increase in air pollutant concentrations with different lag days by season.

Table S6 Relative risk (95% CI) of ALRI, pneumonia and bronchiolitis hospitalizations associated with a 10 µg/m^3^ increase in air pollutant concentrations in single and two-pollutant models.

Table S7 Relative risk (95% CI) in hospital admissions for ALRI, pneumonia and bronchiolitis associated with a 10μg/m^3^ increase in air pollutant concentrations in sensitivity analyses.

Table S1 Spearman correlation between air pollutions and meteorological factors in Lanzhou, China, 2014–2020.

|  | **PM_2.5_** | **PM_10_** | **SO_2_** | **NO_2_** | **Temperature** | **Relative humidity** |
| --- | --- | --- | --- | --- | --- | --- |
| PM_2.5_ | 1.00 |  |  |  |  |  |
| PM_10_ | 0.85* | 1.00 |  |  |  |  |
| SO_2_ | 0.68* | 0.60* | 1.00 |  |  |  |
| NO_2_ | 0.47* | 0.45* | 0.53 * | 1.00 |  |  |
| Temperature | -0.50* | -0.34* | -0.60* | -0.28* | 1.00 |  |
| Relative humidity | -0.16* | -0.42* | -0.26* | -0.17* | -0.03* | 1.00 |

*: *P* ＜ 0. 01.

Table S2 Relative risk (95% CI) of single-pollutant model results in hospital admissions with ALRI, pneumonia and bronchiolitis associated with a 10 µg/m^3^ increase in air pollutant concentrations with different lag days.

| Variables | Lags | PM_2.5_ | | | PM_10_ | | | SO_2_ | | | NO_2_ | | |
| --- | --- | --- | --- | --- | --- | --- | --- | --- | --- | --- | --- | --- | --- |
|  |  | RR | 95%CI | | RR | 95%CI | | RR | 95%CI | | RR | 95%CI | |
|  |  |  | lower | upper |  | lower | upper |  | lower | upper |  | lower | upper |
| ALRI |  |  |  |  |  |  |  |  |  |  |  |  |  |
|  | Lag0 | 1.021 | 1.012 | 1.029 | 1.004 | 1.002 | 1.006 | 1.025 | 1.004 | 1.048 | 1.023 | 1.012 | 1.035 |
|  | Lag1 | 1.009 | 1.000 | 1.018 | 1.001 | 0.999 | 1.004 | 1.038 | 1.012 | 1.064 | 1.020 | 1.007 | 1.035 |
|  | Lag2 | 1.009 | 1.003 | 1.015 | 1.003 | 1.000 | 1.005 | 1.019 | 0.994 | 1.044 | 1.019 | 1.005 | 1.034 |
|  | Lag3 | 1.009 | 1.005 | 1.013 | 1.003 | 1.000 | 1.005 | 1.024 | 0.999 | 1.050 | 1.014 | 1.000 | 1.028 |
|  | Lag4 | 1.008 | 1.004 | 1.012 | 1.001 | 0.999 | 1.004 | 1.010 | 0.986 | 1.035 | 1.016 | 1.002 | 1.030 |
|  | Lag5 | 1.009 | 1.005 | 1.013 | 1.002 | 0.999 | 1.004 | 1.022 | 0.998 | 1.048 | 1.015 | 1.001 | 1.029 |
|  | Lag6 | 1.010 | 1.007 | 1.013 | 1.002 | 0.999 | 1.004 | 1.029 | 1.004 | 1.054 | 1.023 | 1.009 | 1.037 |
|  | Lag7 | 1.011 | 1.005 | 1.017 | 1.002 | 1.000 | 1.004 | 1.006 | 0.984 | 1.028 | 1.009 | 0.998 | 1.021 |
|  | Lag0-1 | 1.030 | 1.021 | 1.039 | 1.005 | 1.003 | 1.008 | 1.064 | 1.039 | 1.090 | 1.044 | 1.031 | 1.058 |
|  | Lag0-2 | 1.040 | 1.031 | 1.049 | 1.008 | 1.006 | 1.011 | 1.084 | 1.056 | 1.113 | 1.064 | 1.049 | 1.080 |
|  | Lag0-3 | 1.049 | 1.038 | 1.059 | 1.011 | 1.008 | 1.014 | 1.110 | 1.079 | 1.142 | 1.079 | 1.062 | 1.096 |
|  | Lag0-4 | 1.057 | 1.046 | 1.069 | 1.012 | 1.009 | 1.015 | 1.121 | 1.088 | 1.155 | 1.096 | 1.078 | 1.115 |
|  | Lag0-5 | 1.067 | 1.054 | 1.079 | 1.014 | 1.010 | 1.017 | 1.146 | 1.112 | 1.182 | 1.113 | 1.094 | 1.132 |
|  | Lag0-6 | 1.077 | 1.064 | 1.090 | 1.016 | 1.012 | 1.019 | 1.179 | 1.143 | 1.216 | 1.138 | 1.118 | 1.159 |
|  | Lag0-7 | 1.089 | 1.075 | 1.103 | 1.018 | 1.014 | 1.021 | 1.186 | 1.154 | 1.219 | 1.149 | 1.130 | 1.168 |
| pneumonia |  |  |  |  |  |  |  |  |  |  |  |  |  |
|  | lag0 | 1.021 | 1.012 | 1.030 | 1.004 | 1.002 | 1.007 | 1.024 | 1.000 | 1.047 | 1.022 | 1.009 | 1.034 |
|  | lag1 | 1.010 | 1.000 | 1.020 | 1.001 | 0.999 | 1.004 | 1.041 | 1.014 | 1.069 | 1.025 | 1.010 | 1.040 |
|  | lag2 | 1.009 | 1.003 | 1.016 | 1.003 | 1.001 | 1.006 | 1.020 | 0.993 | 1.047 | 1.018 | 1.003 | 1.033 |
|  | lag3 | 1.009 | 1.004 | 1.013 | 1.003 | 1.000 | 1.005 | 1.018 | 0.992 | 1.046 | 1.013 | 0.998 | 1.028 |
|  | lag4 | 1.009 | 1.004 | 1.013 | 1.001 | 0.999 | 1.004 | 1.014 | 0.988 | 1.041 | 1.016 | 1.002 | 1.031 |
|  | Lag5 | 1.009 | 1.005 | 1.014 | 1.002 | 0.999 | 1.004 | 1.018 | 0.992 | 1.045 | 1.016 | 1.001 | 1.031 |
|  | Lag6 | 1.011 | 1.008 | 1.014 | 1.002 | 0.999 | 1.004 | 1.032 | 1.005 | 1.059 | 1.023 | 1.009 | 1.038 |
|  | Lag7 | 1.012 | 1.006 | 1.019 | 1.002 | 1.000 | 1.004 | 1.008 | 0.985 | 1.032 | 1.012 | 1.000 | 1.025 |
|  | Lag0-1 | 1.031 | 1.022 | 1.041 | 1.006 | 1.003 | 1.008 | 1.065 | 1.038 | 1.093 | 1.047 | 1.033 | 1.061 |
|  | Lag0-2 | 1.041 | 1.031 | 1.051 | 1.009 | 1.006 | 1.012 | 1.087 | 1.056 | 1.118 | 1.066 | 1.050 | 1.082 |
|  | Lag0-3 | 1.050 | 1.039 | 1.061 | 1.011 | 1.008 | 1.014 | 1.106 | 1.073 | 1.140 | 1.080 | 1.063 | 1.098 |
|  | Lag0-4 | 1.059 | 1.047 | 1.071 | 1.013 | 1.009 | 1.016 | 1.122 | 1.087 | 1.158 | 1.098 | 1.079 | 1.117 |
|  | Lag0-5 | 1.069 | 1.056 | 1.082 | 1.014 | 1.011 | 1.018 | 1.142 | 1.106 | 1.180 | 1.115 | 1.095 | 1.136 |
|  | Lag0-6 | 1.080 | 1.067 | 1.094 | 1.016 | 1.012 | 1.020 | 1.178 | 1.140 | 1.218 | 1.141 | 1.120 | 1.163 |
|  | Lag0-7 | 1.094 | 1.079 | 1.109 | 1.018 | 1.014 | 1.021 | 1.188 | 1.154 | 1.224 | 1.155 | 1.135 | 1.176 |
| bronchiolitis |  |  |  |  |  |  |  |  |  |  |  |  |  |
|  | lag0 | 1.020 | 1.008 | 1.032 | 1.003 | 1.000 | 1.006 | 1.033 | 1.001 | 1.066 | 1.027 | 1.008 | 1.045 |
|  | lag1 | 1.006 | 0.993 | 1.019 | 1.001 | 0.998 | 1.005 | 1.025 | 0.989 | 1.063 | 1.002 | 0.982 | 1.024 |
|  | lag2 | 1.008 | 0.999 | 1.017 | 1.001 | 0.997 | 1.005 | 1.015 | 0.980 | 1.053 | 1.023 | 1.002 | 1.045 |
|  | lag3 | 1.008 | 1.002 | 1.014 | 1.003 | 1.000 | 1.007 | 1.044 | 1.007 | 1.083 | 1.014 | 0.993 | 1.036 |
|  | lag4 | 1.007 | 1.001 | 1.013 | 1.002 | 0.998 | 1.005 | 0.996 | 0.961 | 1.032 | 1.014 | 0.993 | 1.035 |
|  | Lag5 | 1.006 | 1.001 | 1.012 | 1.002 | 0.998 | 1.005 | 1.039 | 1.003 | 1.077 | 1.009 | 0.988 | 1.030 |
|  | Lag6 | 1.006 | 1.001 | 1.010 | 1.002 | 0.998 | 1.006 | 1.020 | 0.985 | 1.057 | 1.021 | 1.000 | 1.043 |
|  | Lag7 | 1.005 | 0.997 | 1.014 | 1.001 | 0.998 | 1.005 | 0.994 | 0.963 | 1.026 | 0.994 | 0.976 | 1.012 |
|  | Lag0-1 | 1.026 | 1.013 | 1.039 | 1.004 | 1.001 | 1.008 | 1.059 | 1.022 | 1.097 | 1.029 | 1.008 | 1.050 |
|  | Lag0-2 | 1.034 | 1.021 | 1.047 | 1.005 | 1.001 | 1.009 | 1.075 | 1.034 | 1.118 | 1.053 | 1.030 | 1.077 |
|  | Lag0-3 | 1.042 | 1.027 | 1.057 | 1.009 | 1.004 | 1.013 | 1.123 | 1.077 | 1.170 | 1.068 | 1.043 | 1.094 |
|  | Lag0-4 | 1.049 | 1.034 | 1.065 | 1.010 | 1.006 | 1.015 | 1.118 | 1.071 | 1.167 | 1.082 | 1.055 | 1.110 |
|  | Lag0-5 | 1.056 | 1.039 | 1.073 | 1.012 | 1.007 | 1.017 | 1.161 | 1.111 | 1.214 | 1.092 | 1.064 | 1.121 |
|  | Lag0-6 | 1.062 | 1.044 | 1.080 | 1.014 | 1.009 | 1.019 | 1.185 | 1.133 | 1.239 | 1.115 | 1.085 | 1.146 |
|  | Lag0-7 | 1.067 | 1.048 | 1.086 | 1.015 | 1.010 | 1.020 | 1.177 | 1.132 | 1.225 | 1.108 | 1.081 | 1.136 |

Table S3 Relative risk (95% CI) of single-pollutant model results in hospital admissions with ALRI, pneumonia and bronchiolitis associated with a 10 µg/m^3^ increase in air pollutant concentrations with different lag days by gender.

| Variables | Gender | Lags | PM_2.5_ | | | PM_10_ | | | SO_2_ | | | NO_2_ | | |
| --- | --- | --- | --- | --- | --- | --- | --- | --- | --- | --- | --- | --- | --- | --- |
|  |  |  | RR | 95%CI | | RR | 95%CI | | RR | 95%CI | | RR | 95%CI | |
|  |  |  |  | lower | upper |  | lower | upper |  | lower | upper |  | lower | upper |
| ALRI |  |  |  |  |  |  |  |  |  |  |  |  |  |  |
|  | Male |  |  |  |  |  |  |  |  |  |  |  |  |  |
|  |  | Lag0 | 1.024 | 1.014 | 1.033 | 1.005 | 1.003 | 1.007 | 1.030 | 1.006 | 1.054 | 1.023 | 1.010 | 1.036 |
|  |  | Lag1 | 1.007 | 0.997 | 1.017 | 1.000 | 0.998 | 1.003 | 1.033 | 1.005 | 1.061 | 1.022 | 1.007 | 1.038 |
|  |  | Lag2 | 1.009 | 1.002 | 1.015 | 1.002 | 1.000 | 1.005 | 1.020 | 0.992 | 1.047 | 1.019 | 1.003 | 1.034 |
|  |  | Lag3 | 1.009 | 1.005 | 1.014 | 1.003 | 1.000 | 1.005 | 1.026 | 0.998 | 1.054 | 1.010 | 0.995 | 1.026 |
|  |  | Lag4 | 1.009 | 1.005 | 1.014 | 1.001 | 0.999 | 1.004 | 1.008 | 0.981 | 1.035 | 1.014 | 0.999 | 1.030 |
|  |  | Lag5 | 1.009 | 1.005 | 1.013 | 1.002 | 0.999 | 1.005 | 1.025 | 0.998 | 1.052 | 1.013 | 0.997 | 1.028 |
|  |  | Lag6 | 1.009 | 1.006 | 1.013 | 1.002 | 0.999 | 1.005 | 1.025 | 0.998 | 1.053 | 1.025 | 1.009 | 1.040 |
|  |  | Lag7 | 1.010 | 1.003 | 1.016 | 1.002 | 0.999 | 1.004 | 1.009 | 0.985 | 1.033 | 1.011 | 0.997 | 1.024 |
|  |  | Lag0-1 | 1.030 | 1.021 | 1.040 | 1.005 | 1.003 | 1.008 | 1.063 | 1.036 | 1.092 | 1.046 | 1.031 | 1.060 |
|  |  | Lag0-2 | 1.039 | 1.029 | 1.049 | 1.008 | 1.005 | 1.011 | 1.084 | 1.053 | 1.116 | 1.065 | 1.048 | 1.082 |
|  |  | Lag0-3 | 1.049 | 1.038 | 1.060 | 1.010 | 1.007 | 1.013 | 1.112 | 1.078 | 1.147 | 1.076 | 1.058 | 1.094 |
|  |  | Lag0-4 | 1.058 | 1.046 | 1.070 | 1.012 | 1.008 | 1.015 | 1.121 | 1.085 | 1.157 | 1.092 | 1.072 | 1.111 |
|  |  | Lag0-5 | 1.068 | 1.055 | 1.081 | 1.014 | 1.010 | 1.017 | 1.148 | 1.111 | 1.187 | 1.105 | 1.084 | 1.126 |
|  |  | Lag0-6 | 1.078 | 1.064 | 1.092 | 1.016 | 1.012 | 1.019 | 1.177 | 1.138 | 1.217 | 1.132 | 1.110 | 1.155 |
|  |  | Lag0-7 | 1.088 | 1.074 | 1.103 | 1.017 | 1.014 | 1.021 | 1.188 | 1.153 | 1.224 | 1.144 | 1.124 | 1.165 |
|  | Female |  |  |  |  |  |  |  |  |  |  |  |  |  |
|  |  | lag0 | 1.017 | 1.007 | 1.028 | 1.003 | 1.001 | 1.006 | 1.019 | 0.993 | 1.045 | 1.024 | 1.010 | 1.038 |
|  |  | lag1 | 1.013 | 1.002 | 1.024 | 1.002 | 0.999 | 1.005 | 1.045 | 1.014 | 1.077 | 1.018 | 1.001 | 1.035 |
|  |  | lag2 | 1.010 | 1.002 | 1.018 | 1.003 | 1.001 | 1.006 | 1.018 | 0.988 | 1.049 | 1.020 | 1.004 | 1.037 |
|  |  | lag3 | 1.008 | 1.003 | 1.013 | 1.003 | 1.000 | 1.006 | 1.022 | 0.992 | 1.053 | 1.018 | 1.001 | 1.036 |
|  |  | lag4 | 1.007 | 1.002 | 1.012 | 1.001 | 0.998 | 1.004 | 1.013 | 0.984 | 1.044 | 1.018 | 1.002 | 1.036 |
|  |  | Lag5 | 1.008 | 1.003 | 1.013 | 1.001 | 0.998 | 1.004 | 1.019 | 0.990 | 1.049 | 1.019 | 1.002 | 1.036 |
|  |  | Lag6 | 1.010 | 1.007 | 1.014 | 1.002 | 0.999 | 1.005 | 1.034 | 1.005 | 1.065 | 1.021 | 1.004 | 1.038 |
|  |  | Lag7 | 1.013 | 1.006 | 1.021 | 1.002 | 1.000 | 1.005 | 1.001 | 0.975 | 1.027 | 1.008 | 0.994 | 1.022 |
|  |  | Lag0-1 | 1.030 | 1.019 | 1.041 | 1.005 | 1.003 | 1.008 | 1.065 | 1.034 | 1.096 | 1.042 | 1.026 | 1.059 |
|  |  | Lag0-2 | 1.040 | 1.029 | 1.051 | 1.009 | 1.006 | 1.012 | 1.084 | 1.050 | 1.119 | 1.064 | 1.045 | 1.082 |
|  |  | Lag0-3 | 1.048 | 1.036 | 1.061 | 1.012 | 1.008 | 1.015 | 1.107 | 1.070 | 1.146 | 1.083 | 1.063 | 1.103 |
|  |  | Lag0-4 | 1.056 | 1.043 | 1.069 | 1.013 | 1.009 | 1.017 | 1.122 | 1.083 | 1.163 | 1.103 | 1.081 | 1.125 |
|  |  | Lag0-5 | 1.064 | 1.050 | 1.079 | 1.014 | 1.010 | 1.018 | 1.143 | 1.102 | 1.186 | 1.124 | 1.101 | 1.147 |
|  |  | Lag0-6 | 1.075 | 1.060 | 1.091 | 1.015 | 1.011 | 1.020 | 1.183 | 1.140 | 1.228 | 1.147 | 1.123 | 1.172 |
|  |  | Lag0-7 | 1.090 | 1.073 | 1.106 | 1.018 | 1.014 | 1.022 | 1.184 | 1.145 | 1.224 | 1.156 | 1.133 | 1.179 |
| pneumonia |  |  |  |  |  |  |  |  |  |  |  |  |  |  |
|  | Male |  |  |  |  |  |  |  |  |  |  |  |  |  |
|  |  | lag0 | 1.024 | 1.014 | 1.034 | 1.005 | 1.003 | 1.007 | 1.024 | 0.999 | 1.050 | 1.020 | 1.006 | 1.034 |
|  |  | lag1 | 1.007 | 0.997 | 1.018 | 1.000 | 0.997 | 1.003 | 1.036 | 1.006 | 1.066 | 1.026 | 1.009 | 1.042 |
|  |  | lag2 | 1.008 | 1.001 | 1.016 | 1.003 | 1.000 | 1.006 | 1.023 | 0.994 | 1.053 | 1.019 | 1.003 | 1.036 |
|  |  | lag3 | 1.009 | 1.004 | 1.014 | 1.003 | 1.000 | 1.005 | 1.018 | 0.989 | 1.048 | 1.010 | 0.994 | 1.027 |
|  |  | lag4 | 1.010 | 1.005 | 1.014 | 1.001 | 0.999 | 1.004 | 1.010 | 0.982 | 1.040 | 1.013 | 0.996 | 1.029 |
|  |  | Lag5 | 1.010 | 1.006 | 1.015 | 1.002 | 1.000 | 1.005 | 1.026 | 0.997 | 1.056 | 1.015 | 0.999 | 1.032 |
|  |  | Lag6 | 1.011 | 1.008 | 1.014 | 1.002 | 0.999 | 1.005 | 1.025 | 0.997 | 1.055 | 1.024 | 1.008 | 1.041 |
|  |  | Lag7 | 1.012 | 1.005 | 1.019 | 1.002 | 0.999 | 1.005 | 1.014 | 0.989 | 1.040 | 1.016 | 1.002 | 1.031 |
|  |  | Lag0-1 | 1.031 | 1.021 | 1.042 | 1.005 | 1.003 | 1.008 | 1.060 | 1.031 | 1.091 | 1.046 | 1.030 | 1.062 |
|  |  | Lag0-2 | 1.040 | 1.029 | 1.050 | 1.008 | 1.005 | 1.011 | 1.085 | 1.052 | 1.119 | 1.066 | 1.048 | 1.084 |
|  |  | Lag0-3 | 1.049 | 1.037 | 1.062 | 1.011 | 1.008 | 1.014 | 1.105 | 1.069 | 1.142 | 1.077 | 1.058 | 1.097 |
|  |  | Lag0-4 | 1.059 | 1.047 | 1.072 | 1.012 | 1.009 | 1.016 | 1.117 | 1.079 | 1.156 | 1.091 | 1.070 | 1.112 |
|  |  | Lag0-5 | 1.070 | 1.056 | 1.085 | 1.015 | 1.011 | 1.019 | 1.146 | 1.106 | 1.188 | 1.107 | 1.085 | 1.130 |
|  |  | Lag0-6 | 1.082 | 1.067 | 1.097 | 1.016 | 1.012 | 1.020 | 1.175 | 1.133 | 1.219 | 1.134 | 1.110 | 1.158 |
|  |  | Lag0-7 | 1.095 | 1.079 | 1.111 | 1.018 | 1.014 | 1.022 | 1.192 | 1.154 | 1.231 | 1.153 | 1.130 | 1.175 |
|  | Female |  |  |  |  |  |  |  |  |  |  |  |  |  |
|  |  | lag0 | 1.017 | 1.006 | 1.029 | 1.003 | 1.001 | 1.006 | 1.023 | 0.995 | 1.052 | 1.024 | 1.009 | 1.040 |
|  |  | lag1 | 1.014 | 1.002 | 1.026 | 1.003 | 1.000 | 1.005 | 1.049 | 1.015 | 1.083 | 1.024 | 1.006 | 1.042 |
|  |  | lag2 | 1.011 | 1.002 | 1.019 | 1.004 | 1.001 | 1.007 | 1.015 | 0.983 | 1.048 | 1.017 | 0.999 | 1.035 |
|  |  | lag3 | 1.008 | 1.003 | 1.013 | 1.003 | 0.999 | 1.006 | 1.018 | 0.986 | 1.052 | 1.017 | 0.999 | 1.036 |
|  |  | lag4 | 1.007 | 1.002 | 1.012 | 1.001 | 0.998 | 1.004 | 1.019 | 0.987 | 1.052 | 1.022 | 1.004 | 1.040 |
|  |  | Lag5 | 1.008 | 1.003 | 1.013 | 1.000 | 0.997 | 1.004 | 1.007 | 0.975 | 1.039 | 1.016 | 0.999 | 1.035 |
|  |  | Lag6 | 1.010 | 1.007 | 1.014 | 1.002 | 0.999 | 1.005 | 1.040 | 1.008 | 1.074 | 1.022 | 1.004 | 1.041 |
|  |  | Lag7 | 1.013 | 1.005 | 1.022 | 1.002 | 0.999 | 1.005 | 1.000 | 0.973 | 1.029 | 1.007 | 0.991 | 1.022 |
|  |  | Lag0-1 | 1.032 | 1.020 | 1.044 | 1.006 | 1.003 | 1.009 | 1.073 | 1.040 | 1.107 | 1.048 | 1.031 | 1.066 |
|  |  | Lag0-2 | 1.043 | 1.031 | 1.055 | 1.009 | 1.006 | 1.013 | 1.089 | 1.052 | 1.127 | 1.066 | 1.046 | 1.086 |
|  |  | Lag0-3 | 1.051 | 1.037 | 1.065 | 1.012 | 1.008 | 1.016 | 1.109 | 1.069 | 1.150 | 1.084 | 1.063 | 1.106 |
|  |  | Lag0-4 | 1.058 | 1.044 | 1.073 | 1.013 | 1.009 | 1.017 | 1.130 | 1.087 | 1.174 | 1.108 | 1.084 | 1.132 |
|  |  | Lag0-5 | 1.067 | 1.051 | 1.083 | 1.013 | 1.009 | 1.018 | 1.138 | 1.093 | 1.184 | 1.126 | 1.101 | 1.152 |
|  |  | Lag0-6 | 1.078 | 1.061 | 1.095 | 1.015 | 1.011 | 1.020 | 1.183 | 1.137 | 1.232 | 1.151 | 1.125 | 1.178 |
|  |  | Lag0-7 | 1.092 | 1.074 | 1.110 | 1.017 | 1.013 | 1.022 | 1.184 | 1.142 | 1.227 | 1.159 | 1.134 | 1.184 |
| bronchiolitis |  |  |  |  |  |  |  |  |  |  |  |  |  |  |
|  | Male |  |  |  |  |  |  |  |  |  |  |  |  |  |
|  |  | lag0 | 1.023 | 1.009 | 1.037 | 1.003 | 0.999 | 1.007 | 1.053 | 1.015 | 1.093 | 1.032 | 1.010 | 1.054 |
|  |  | lag1 | 1.005 | 0.990 | 1.020 | 1.002 | 0.997 | 1.006 | 1.020 | 0.978 | 1.065 | 1.008 | 0.983 | 1.034 |
|  |  | lag2 | 1.009 | 0.998 | 1.019 | 1.000 | 0.996 | 1.005 | 1.007 | 0.965 | 1.051 | 1.016 | 0.991 | 1.042 |
|  |  | lag3 | 1.009 | 1.002 | 1.016 | 1.003 | 0.998 | 1.007 | 1.051 | 1.006 | 1.097 | 1.009 | 0.984 | 1.035 |
|  |  | lag4 | 1.007 | 1.000 | 1.014 | 1.001 | 0.997 | 1.006 | 0.998 | 0.956 | 1.041 | 1.020 | 0.994 | 1.046 |
|  |  | Lag5 | 1.005 | 0.998 | 1.011 | 1.001 | 0.997 | 1.006 | 1.019 | 0.976 | 1.063 | 0.999 | 0.974 | 1.024 |
|  |  | Lag6 | 1.003 | 0.998 | 1.008 | 1.002 | 0.998 | 1.007 | 1.024 | 0.982 | 1.068 | 1.026 | 1.001 | 1.052 |
|  |  | Lag7 | 1.001 | 0.991 | 1.011 | 1.000 | 0.996 | 1.004 | 0.989 | 0.952 | 1.027 | 0.984 | 0.963 | 1.006 |
|  |  | Lag0-1 | 1.027 | 1.012 | 1.043 | 1.005 | 1.001 | 1.009 | 1.074 | 1.030 | 1.121 | 1.041 | 1.016 | 1.066 |
|  |  | Lag0-2 | 1.036 | 1.021 | 1.052 | 1.005 | 1.000 | 1.010 | 1.082 | 1.033 | 1.133 | 1.058 | 1.030 | 1.086 |
|  |  | Lag0-3 | 1.046 | 1.028 | 1.063 | 1.008 | 1.003 | 1.013 | 1.137 | 1.083 | 1.194 | 1.067 | 1.037 | 1.098 |
|  |  | Lag0-4 | 1.053 | 1.034 | 1.071 | 1.009 | 1.003 | 1.015 | 1.135 | 1.078 | 1.194 | 1.089 | 1.056 | 1.122 |
|  |  | Lag0-5 | 1.058 | 1.037 | 1.078 | 1.010 | 1.004 | 1.016 | 1.156 | 1.097 | 1.218 | 1.088 | 1.054 | 1.122 |
|  |  | Lag0-6 | 1.061 | 1.039 | 1.082 | 1.012 | 1.006 | 1.019 | 1.184 | 1.122 | 1.249 | 1.116 | 1.080 | 1.152 |
|  |  | Lag0-7 | 1.061 | 1.039 | 1.084 | 1.013 | 1.007 | 1.019 | 1.170 | 1.117 | 1.226 | 1.098 | 1.066 | 1.131 |
|  | Female |  |  |  |  |  |  |  |  |  |  |  |  |  |
|  |  | lag0 | 1.016 | 0.999 | 1.033 | 1.003 | 0.999 | 1.007 | 1.002 | 0.958 | 1.047 | 1.018 | 0.993 | 1.044 |
|  |  | lag1 | 1.007 | 0.989 | 1.026 | 1.001 | 0.995 | 1.006 | 1.033 | 0.981 | 1.087 | 0.993 | 0.964 | 1.023 |
|  |  | lag2 | 1.007 | 0.994 | 1.020 | 1.002 | 0.997 | 1.007 | 1.029 | 0.977 | 1.083 | 1.034 | 1.004 | 1.066 |
|  |  | lag3 | 1.007 | 0.998 | 1.015 | 1.004 | 0.999 | 1.009 | 1.034 | 0.982 | 1.089 | 1.022 | 0.991 | 1.053 |
|  |  | lag4 | 1.007 | 0.999 | 1.015 | 1.002 | 0.997 | 1.007 | 0.992 | 0.943 | 1.044 | 1.005 | 0.975 | 1.035 |
|  |  | Lag5 | 1.008 | 1.000 | 1.016 | 1.003 | 0.998 | 1.008 | 1.071 | 1.018 | 1.127 | 1.025 | 0.995 | 1.055 |
|  |  | Lag6 | 1.010 | 1.004 | 1.016 | 1.001 | 0.996 | 1.006 | 1.014 | 0.965 | 1.067 | 1.014 | 0.985 | 1.045 |
|  |  | Lag7 | 1.012 | 1.000 | 1.024 | 1.003 | 0.999 | 1.008 | 1.002 | 0.958 | 1.048 | 1.008 | 0.983 | 1.034 |
|  |  | Lag0-1 | 1.023 | 1.005 | 1.042 | 1.004 | 0.999 | 1.009 | 1.035 | 0.984 | 1.088 | 1.011 | 0.983 | 1.040 |
|  |  | Lag0-2 | 1.030 | 1.012 | 1.049 | 1.006 | 1.000 | 1.012 | 1.064 | 1.007 | 1.125 | 1.046 | 1.013 | 1.080 |
|  |  | Lag0-3 | 1.037 | 1.016 | 1.059 | 1.010 | 1.004 | 1.016 | 1.101 | 1.038 | 1.167 | 1.068 | 1.033 | 1.105 |
|  |  | Lag0-4 | 1.044 | 1.022 | 1.067 | 1.012 | 1.006 | 1.019 | 1.092 | 1.027 | 1.161 | 1.073 | 1.036 | 1.113 |
|  |  | Lag0-5 | 1.053 | 1.029 | 1.078 | 1.015 | 1.008 | 1.022 | 1.170 | 1.098 | 1.245 | 1.100 | 1.059 | 1.142 |
|  |  | Lag0-6 | 1.063 | 1.038 | 1.089 | 1.016 | 1.008 | 1.023 | 1.186 | 1.113 | 1.264 | 1.116 | 1.073 | 1.159 |
|  |  | Lag0-7 | 1.075 | 1.049 | 1.103 | 1.019 | 1.012 | 1.026 | 1.189 | 1.124 | 1.258 | 1.125 | 1.086 | 1.165 |

Table S4 Relative risk (95% CI) of single-pollutant model results in hospital admissions with ALRI, pneumonia and bronchiolitis associated with a 10µg/m^3^ increase in air pollutant concentrations with different lag days by age.

| Variables | Age | Lags | PM_2.5_ | | | PM_10_ | | | SO_2_ | | | NO_2_ | | |
| --- | --- | --- | --- | --- | --- | --- | --- | --- | --- | --- | --- | --- | --- | --- |
|  |  |  | RR | 95%CI | | RR | 95%CI | | RR | 95%CI | | RR | 95%CI | |
|  |  |  |  | lower | upper |  | lower | upper |  | lower | upper |  | lower | upper |
| ALRI |  |  |  |  |  |  |  |  |  |  |  |  |  |  |
|  | <5 y |  |  |  |  |  |  |  |  |  |  |  |  |  |
|  |  | Lag0 | 1.021 | 1.013 | 1.030 | 1.004 | 1.002 | 1.007 | 1.026 | 1.004 | 1.050 | 1.024 | 1.011 | 1.036 |
|  |  | Lag1 | 1.009 | 1.000 | 1.019 | 1.001 | 0.999 | 1.004 | 1.030 | 1.003 | 1.056 | 1.016 | 1.002 | 1.031 |
|  |  | Lag2 | 1.010 | 1.004 | 1.016 | 1.003 | 1.000 | 1.005 | 1.017 | 0.991 | 1.044 | 1.019 | 1.005 | 1.034 |
|  |  | Lag3 | 1.009 | 1.005 | 1.014 | 1.003 | 1.001 | 1.006 | 1.021 | 0.995 | 1.048 | 1.011 | 0.996 | 1.026 |
|  |  | Lag4 | 1.009 | 1.005 | 1.013 | 1.001 | 0.999 | 1.004 | 1.012 | 0.987 | 1.039 | 1.016 | 1.001 | 1.031 |
|  |  | Lag5 | 1.009 | 1.005 | 1.013 | 1.001 | 0.999 | 1.004 | 1.020 | 0.994 | 1.046 | 1.013 | 0.999 | 1.028 |
|  |  | Lag6 | 1.011 | 1.008 | 1.014 | 1.002 | 1.000 | 1.005 | 1.024 | 0.998 | 1.050 | 1.021 | 1.006 | 1.036 |
|  |  | Lag7 | 1.013 | 1.006 | 1.019 | 1.002 | 1.000 | 1.004 | 1.013 | 0.991 | 1.036 | 1.011 | 0.998 | 1.023 |
|  |  | Lag0-1 | 1.031 | 1.021 | 1.040 | 1.006 | 1.003 | 1.008 | 1.057 | 1.030 | 1.084 | 1.040 | 1.026 | 1.055 |
|  |  | Lag0-2 | 1.041 | 1.032 | 1.050 | 1.008 | 1.006 | 1.011 | 1.075 | 1.046 | 1.105 | 1.061 | 1.045 | 1.077 |
|  |  | Lag0-3 | 1.051 | 1.040 | 1.062 | 1.012 | 1.009 | 1.015 | 1.098 | 1.066 | 1.131 | 1.072 | 1.055 | 1.090 |
|  |  | Lag0-4 | 1.060 | 1.049 | 1.071 | 1.013 | 1.010 | 1.016 | 1.112 | 1.078 | 1.147 | 1.089 | 1.071 | 1.108 |
|  |  | Lag0-5 | 1.070 | 1.058 | 1.083 | 1.015 | 1.011 | 1.018 | 1.134 | 1.098 | 1.171 | 1.104 | 1.084 | 1.124 |
|  |  | Lag0-6 | 1.082 | 1.069 | 1.095 | 1.017 | 1.013 | 1.020 | 1.160 | 1.123 | 1.199 | 1.127 | 1.106 | 1.148 |
|  |  | Lag0-7 | 1.095 | 1.081 | 1.109 | 1.019 | 1.015 | 1.022 | 1.176 | 1.142 | 1.210 | 1.139 | 1.119 | 1.159 |
|  | 5-14 y |  |  |  |  |  |  |  |  |  |  |  |  |  |
|  |  | lag0 | 1.020 | 1.006 | 1.033 | 1.003 | 1.000 | 1.006 | 1.020 | 0.987 | 1.054 | 1.021 | 1.003 | 1.039 |
|  |  | lag1 | 1.010 | 0.995 | 1.024 | 1.001 | 0.997 | 1.005 | 1.067 | 1.027 | 1.108 | 1.036 | 1.014 | 1.058 |
|  |  | lag2 | 1.006 | 0.996 | 1.016 | 1.004 | 1.000 | 1.007 | 1.024 | 0.985 | 1.064 | 1.019 | 0.997 | 1.041 |
|  |  | lag3 | 1.006 | 0.999 | 1.012 | 1.001 | 0.997 | 1.005 | 1.034 | 0.995 | 1.075 | 1.024 | 1.002 | 1.046 |
|  |  | lag4 | 1.006 | 1.000 | 1.013 | 1.001 | 0.997 | 1.005 | 1.003 | 0.966 | 1.042 | 1.017 | 0.995 | 1.038 |
|  |  | Lag5 | 1.006 | 1.000 | 1.012 | 1.002 | 0.998 | 1.006 | 1.031 | 0.993 | 1.071 | 1.021 | 0.999 | 1.042 |
|  |  | Lag6 | 1.006 | 1.001 | 1.011 | 1.000 | 0.996 | 1.004 | 1.052 | 1.013 | 1.092 | 1.031 | 1.009 | 1.053 |
|  |  | Lag7 | 1.006 | 0.996 | 1.016 | 1.002 | 0.999 | 1.006 | 0.977 | 0.945 | 1.010 | 1.006 | 0.987 | 1.024 |
|  |  | Lag0-1 | 1.030 | 1.015 | 1.044 | 1.004 | 1.000 | 1.007 | 1.088 | 1.048 | 1.129 | 1.057 | 1.036 | 1.078 |
|  |  | Lag0-2 | 1.036 | 1.021 | 1.050 | 1.007 | 1.003 | 1.011 | 1.114 | 1.070 | 1.160 | 1.077 | 1.053 | 1.102 |
|  |  | Lag0-3 | 1.042 | 1.025 | 1.058 | 1.008 | 1.004 | 1.013 | 1.152 | 1.104 | 1.203 | 1.103 | 1.077 | 1.130 |
|  |  | Lag0-4 | 1.048 | 1.031 | 1.066 | 1.009 | 1.004 | 1.014 | 1.156 | 1.105 | 1.209 | 1.121 | 1.093 | 1.150 |
|  |  | Lag0-5 | 1.055 | 1.036 | 1.074 | 1.011 | 1.006 | 1.017 | 1.192 | 1.138 | 1.249 | 1.144 | 1.114 | 1.175 |
|  |  | Lag0-6 | 1.061 | 1.041 | 1.081 | 1.011 | 1.006 | 1.017 | 1.254 | 1.196 | 1.314 | 1.179 | 1.147 | 1.213 |
|  |  | Lag0-7 | 1.067 | 1.046 | 1.089 | 1.013 | 1.008 | 1.019 | 1.225 | 1.174 | 1.277 | 1.186 | 1.156 | 1.217 |
| pneumonia |  |  |  |  |  |  |  |  |  |  |  |  |  |  |
|  | <5 y |  |  |  |  |  |  |  |  |  |  |  |  |  |
|  |  | lag0 | 1.021 | 1.012 | 1.030 | 1.005 | 1.002 | 1.007 | 1.022 | 0.998 | 1.047 | 1.022 | 1.009 | 1.035 |
|  |  | lag1 | 1.011 | 1.001 | 1.021 | 1.002 | 0.999 | 1.004 | 1.033 | 1.005 | 1.061 | 1.020 | 1.005 | 1.036 |
|  |  | lag2 | 1.010 | 1.003 | 1.017 | 1.003 | 1.000 | 1.005 | 1.019 | 0.991 | 1.047 | 1.019 | 1.003 | 1.034 |
|  |  | lag3 | 1.009 | 1.005 | 1.014 | 1.003 | 1.000 | 1.006 | 1.014 | 0.987 | 1.043 | 1.011 | 0.995 | 1.027 |
|  |  | lag4 | 1.009 | 1.005 | 1.014 | 1.001 | 0.998 | 1.004 | 1.016 | 0.988 | 1.044 | 1.015 | 0.999 | 1.031 |
|  |  | Lag5 | 1.010 | 1.006 | 1.015 | 1.002 | 0.999 | 1.004 | 1.017 | 0.990 | 1.045 | 1.015 | 0.999 | 1.030 |
|  |  | Lag6 | 1.012 | 1.009 | 1.015 | 1.002 | 0.999 | 1.005 | 1.024 | 0.996 | 1.052 | 1.021 | 1.005 | 1.036 |
|  |  | Lag7 | 1.014 | 1.007 | 1.021 | 1.002 | 1.000 | 1.004 | 1.018 | 0.994 | 1.043 | 1.016 | 1.002 | 1.029 |
|  |  | Lag0-1 | 1.032 | 1.022 | 1.042 | 1.006 | 1.004 | 1.009 | 1.056 | 1.028 | 1.084 | 1.043 | 1.028 | 1.058 |
|  |  | Lag0-2 | 1.042 | 1.032 | 1.052 | 1.009 | 1.006 | 1.012 | 1.075 | 1.044 | 1.108 | 1.062 | 1.045 | 1.079 |
|  |  | Lag0-3 | 1.052 | 1.040 | 1.063 | 1.012 | 1.009 | 1.015 | 1.091 | 1.057 | 1.126 | 1.074 | 1.055 | 1.092 |
|  |  | Lag0-4 | 1.061 | 1.049 | 1.074 | 1.013 | 1.010 | 1.017 | 1.108 | 1.072 | 1.145 | 1.090 | 1.070 | 1.110 |
|  |  | Lag0-5 | 1.072 | 1.059 | 1.086 | 1.015 | 1.011 | 1.019 | 1.127 | 1.089 | 1.166 | 1.106 | 1.085 | 1.127 |
|  |  | Lag0-6 | 1.085 | 1.071 | 1.099 | 1.017 | 1.013 | 1.021 | 1.154 | 1.114 | 1.194 | 1.128 | 1.106 | 1.151 |
|  |  | Lag0-7 | 1.100 | 1.085 | 1.116 | 1.019 | 1.016 | 1.023 | 1.175 | 1.139 | 1.212 | 1.146 | 1.125 | 1.167 |
|  | 5-14 y |  |  |  |  |  |  |  |  |  |  |  |  |  |
|  |  | lag0 | 1.022 | 1.008 | 1.037 | 1.004 | 1.001 | 1.007 | 1.027 | 0.991 | 1.064 | 1.021 | 1.001 | 1.041 |
|  |  | lag1 | 1.009 | 0.993 | 1.025 | 0.999 | 0.995 | 1.004 | 1.071 | 1.027 | 1.117 | 1.041 | 1.017 | 1.065 |
|  |  | lag2 | 1.007 | 0.996 | 1.018 | 1.005 | 1.001 | 1.008 | 1.024 | 0.982 | 1.068 | 1.017 | 0.994 | 1.041 |
|  |  | lag3 | 1.006 | 0.999 | 1.013 | 1.001 | 0.996 | 1.005 | 1.035 | 0.993 | 1.079 | 1.023 | 0.999 | 1.047 |
|  |  | lag4 | 1.006 | 0.999 | 1.013 | 1.001 | 0.997 | 1.006 | 1.009 | 0.969 | 1.052 | 1.023 | 1.000 | 1.047 |
|  |  | Lag5 | 1.006 | 0.999 | 1.013 | 1.001 | 0.997 | 1.005 | 1.022 | 0.981 | 1.065 | 1.019 | 0.996 | 1.042 |
|  |  | Lag6 | 1.006 | 1.001 | 1.011 | 1.000 | 0.995 | 1.004 | 1.064 | 1.021 | 1.109 | 1.033 | 1.010 | 1.057 |
|  |  | Lag7 | 1.006 | 0.996 | 1.017 | 1.002 | 0.998 | 1.006 | 0.970 | 0.935 | 1.006 | 1.001 | 0.982 | 1.021 |
|  |  | Lag0-1 | 1.031 | 1.016 | 1.047 | 1.003 | 0.999 | 1.007 | 1.100 | 1.056 | 1.145 | 1.063 | 1.040 | 1.086 |
|  |  | Lag0-2 | 1.038 | 1.023 | 1.054 | 1.008 | 1.004 | 1.012 | 1.126 | 1.077 | 1.177 | 1.081 | 1.055 | 1.107 |
|  |  | Lag0-3 | 1.045 | 1.027 | 1.063 | 1.009 | 1.004 | 1.013 | 1.165 | 1.112 | 1.222 | 1.105 | 1.077 | 1.134 |
|  |  | Lag0-4 | 1.051 | 1.032 | 1.070 | 1.010 | 1.005 | 1.015 | 1.176 | 1.120 | 1.236 | 1.131 | 1.100 | 1.162 |
|  |  | Lag0-5 | 1.058 | 1.037 | 1.079 | 1.011 | 1.005 | 1.017 | 1.203 | 1.143 | 1.265 | 1.152 | 1.119 | 1.186 |
|  |  | Lag0-6 | 1.064 | 1.043 | 1.086 | 1.011 | 1.005 | 1.017 | 1.280 | 1.215 | 1.348 | 1.190 | 1.155 | 1.227 |
|  |  | Lag0-7 | 1.071 | 1.048 | 1.094 | 1.013 | 1.007 | 1.019 | 1.241 | 1.186 | 1.299 | 1.192 | 1.159 | 1.226 |
| bronchiolitis |  |  |  |  |  |  |  |  |  |  |  |  |  |  |
|  | <5 y |  |  |  |  |  |  |  |  |  |  |  |  |  |
|  |  | lag0 | 1.023 | 1.010 | 1.036 | 1.004 | 1.001 | 1.008 | 1.045 | 1.009 | 1.082 | 1.029 | 1.008 | 1.049 |
|  |  | lag1 | 1.004 | 0.990 | 1.018 | 1.000 | 0.996 | 1.004 | 1.017 | 0.978 | 1.059 | 0.998 | 0.975 | 1.022 |
|  |  | lag2 | 1.009 | 1.000 | 1.019 | 1.002 | 0.998 | 1.006 | 1.013 | 0.974 | 1.054 | 1.022 | 0.999 | 1.047 |
|  |  | lag3 | 1.009 | 1.003 | 1.015 | 1.003 | 0.999 | 1.007 | 1.048 | 1.007 | 1.090 | 1.010 | 0.986 | 1.034 |
|  |  | lag4 | 1.007 | 1.001 | 1.013 | 1.002 | 0.998 | 1.006 | 1.000 | 0.961 | 1.040 | 1.020 | 0.997 | 1.044 |
|  |  | Lag5 | 1.006 | 1.000 | 1.012 | 1.001 | 0.997 | 1.005 | 1.032 | 0.992 | 1.073 | 1.005 | 0.982 | 1.029 |
|  |  | Lag6 | 1.006 | 1.001 | 1.010 | 1.002 | 0.998 | 1.006 | 1.024 | 0.985 | 1.065 | 1.021 | 0.998 | 1.045 |
|  |  | Lag7 | 1.006 | 0.996 | 1.015 | 1.002 | 0.998 | 1.005 | 0.991 | 0.957 | 1.026 | 0.986 | 0.967 | 1.006 |
|  |  | Lag0-1 | 1.027 | 1.013 | 1.041 | 1.004 | 1.000 | 1.008 | 1.063 | 1.023 | 1.106 | 1.027 | 1.004 | 1.050 |
|  |  | Lag0-2 | 1.036 | 1.022 | 1.051 | 1.006 | 1.001 | 1.010 | 1.077 | 1.032 | 1.125 | 1.050 | 1.024 | 1.076 |
|  |  | Lag0-3 | 1.046 | 1.029 | 1.062 | 1.009 | 1.004 | 1.014 | 1.129 | 1.078 | 1.182 | 1.060 | 1.032 | 1.088 |
|  |  | Lag0-4 | 1.053 | 1.036 | 1.070 | 1.011 | 1.006 | 1.017 | 1.129 | 1.076 | 1.184 | 1.081 | 1.051 | 1.112 |
|  |  | Lag0-5 | 1.060 | 1.041 | 1.079 | 1.012 | 1.007 | 1.018 | 1.164 | 1.109 | 1.223 | 1.087 | 1.055 | 1.119 |
|  |  | Lag0-6 | 1.066 | 1.046 | 1.086 | 1.014 | 1.008 | 1.020 | 1.193 | 1.135 | 1.253 | 1.109 | 1.076 | 1.143 |
|  |  | Lag0-7 | 1.072 | 1.051 | 1.093 | 1.016 | 1.010 | 1.021 | 1.182 | 1.131 | 1.234 | 1.094 | 1.064 | 1.125 |
|  | 5-14 y |  |  |  |  |  |  |  |  |  |  |  |  |  |
|  |  | lag0 | 1.010 | 0.988 | 1.033 | 0.999 | 0.992 | 1.005 | 0.994 | 0.938 | 1.055 | 1.020 | 0.986 | 1.055 |
|  |  | lag1 | 1.012 | 0.988 | 1.037 | 1.006 | 1.000 | 1.013 | 1.050 | 0.981 | 1.124 | 1.016 | 0.976 | 1.058 |
|  |  | lag2 | 1.004 | 0.987 | 1.021 | 0.998 | 0.989 | 1.006 | 1.026 | 0.958 | 1.099 | 1.028 | 0.987 | 1.070 |
|  |  | lag3 | 1.004 | 0.993 | 1.015 | 1.003 | 0.995 | 1.010 | 1.033 | 0.964 | 1.107 | 1.029 | 0.988 | 1.072 |
|  |  | lag4 | 1.006 | 0.995 | 1.017 | 1.000 | 0.992 | 1.007 | 0.982 | 0.918 | 1.052 | 0.992 | 0.953 | 1.032 |
|  |  | Lag5 | 1.006 | 0.996 | 1.017 | 1.006 | 0.999 | 1.012 | 1.063 | 0.994 | 1.137 | 1.022 | 0.982 | 1.063 |
|  |  | Lag6 | 1.005 | 0.997 | 1.013 | 1.001 | 0.993 | 1.008 | 1.009 | 0.943 | 1.079 | 1.022 | 0.983 | 1.063 |
|  |  | Lag7 | 1.004 | 0.987 | 1.020 | 1.001 | 0.994 | 1.008 | 1.000 | 0.943 | 1.062 | 1.021 | 0.986 | 1.056 |
|  |  | Lag0-1 | 1.023 | 0.999 | 1.048 | 1.005 | 0.998 | 1.012 | 1.044 | 0.977 | 1.116 | 1.036 | 0.997 | 1.077 |
|  |  | Lag0-2 | 1.027 | 1.002 | 1.052 | 1.003 | 0.994 | 1.011 | 1.071 | 0.995 | 1.152 | 1.065 | 1.020 | 1.111 |
|  |  | Lag0-3 | 1.031 | 1.003 | 1.060 | 1.005 | 0.997 | 1.014 | 1.107 | 1.024 | 1.196 | 1.096 | 1.047 | 1.147 |
|  |  | Lag0-4 | 1.037 | 1.007 | 1.068 | 1.005 | 0.996 | 1.015 | 1.087 | 1.003 | 1.179 | 1.087 | 1.036 | 1.140 |
|  |  | Lag0-5 | 1.043 | 1.011 | 1.077 | 1.011 | 1.001 | 1.021 | 1.156 | 1.064 | 1.255 | 1.110 | 1.056 | 1.167 |
|  |  | Lag0-6 | 1.049 | 1.015 | 1.084 | 1.012 | 1.001 | 1.022 | 1.166 | 1.072 | 1.268 | 1.135 | 1.078 | 1.195 |
|  |  | Lag0-7 | 1.052 | 1.016 | 1.090 | 1.013 | 1.002 | 1.023 | 1.166 | 1.084 | 1.255 | 1.158 | 1.105 | 1.214 |

Table S5 Relative risk (95% CI) of single-pollutant model results in hospital admissions with ALRI, pneumonia and bronchiolitis associated with a 10 µg/m^3^ increase in air pollutant concentrations with different lag days by season.

| Variables | Season | Lags | PM_2.5_ | | | PM_10_ | | | SO_2_ | | | NO_2_ | | |
| --- | --- | --- | --- | --- | --- | --- | --- | --- | --- | --- | --- | --- | --- | --- |
|  |  |  | RR | 95%CI | | RR | 95%CI | | RR | 95%CI | | RR | 95%CI | |
|  |  |  |  | lower | upper |  | lower | upper |  | lower | upper |  | lower | upper |
| ALRI |  |  |  |  |  |  |  |  |  |  |  |  |  |  |
|  | Warm |  |  |  |  |  |  |  |  |  |  |  |  |  |
|  |  | Lag0 | 1.003 | 0.991 | 1.015 | 0.999 | 0.996 | 1.003 | 1.007 | 0.963 | 1.053 | 0.989 | 0.967 | 1.011 |
|  |  | Lag1 | 1.004 | 0.992 | 1.016 | 1.003 | 1.000 | 1.006 | 1.037 | 0.991 | 1.084 | 1.001 | 0.978 | 1.026 |
|  |  | Lag2 | 1.004 | 0.995 | 1.013 | 1.000 | 0.997 | 1.003 | 1.033 | 0.987 | 1.081 | 1.015 | 0.991 | 1.040 |
|  |  | Lag3 | 1.005 | 0.999 | 1.011 | 1.002 | 0.998 | 1.005 | 1.014 | 0.969 | 1.061 | 1.006 | 0.982 | 1.030 |
|  |  | Lag4 | 1.006 | 1.000 | 1.012 | 1.001 | 0.998 | 1.005 | 0.973 | 0.929 | 1.018 | 0.996 | 0.972 | 1.020 |
|  |  | Lag5 | 1.007 | 1.001 | 1.012 | 1.001 | 0.998 | 1.004 | 1.011 | 0.967 | 1.056 | 1.009 | 0.985 | 1.033 |
|  |  | Lag6 | 1.006 | 1.001 | 1.011 | 1.003 | 0.999 | 1.006 | 1.060 | 1.015 | 1.107 | 1.023 | 0.999 | 1.048 |
|  |  | Lag7 | 1.005 | 0.996 | 1.014 | 1.000 | 0.997 | 1.003 | 1.065 | 1.022 | 1.110 | 1.025 | 1.004 | 1.047 |
|  |  | Lag0-1 | 1.007 | 0.993 | 1.021 | 1.002 | 0.999 | 1.006 | 1.044 | 0.986 | 1.106 | 0.990 | 0.964 | 1.017 |
|  |  | Lag0-2 | 1.011 | 0.996 | 1.026 | 1.003 | 0.999 | 1.007 | 1.079 | 1.011 | 1.151 | 1.005 | 0.975 | 1.036 |
|  |  | Lag0-3 | 1.016 | 0.999 | 1.034 | 1.004 | 1.000 | 1.008 | 1.093 | 1.021 | 1.172 | 1.011 | 0.978 | 1.045 |
|  |  | Lag0-4 | 1.023 | 1.004 | 1.041 | 1.005 | 1.000 | 1.010 | 1.064 | 0.989 | 1.144 | 1.007 | 0.972 | 1.043 |
|  |  | Lag0-5 | 1.029 | 1.009 | 1.050 | 1.006 | 1.001 | 1.011 | 1.075 | 0.997 | 1.159 | 1.015 | 0.978 | 1.054 |
|  |  | Lag0-6 | 1.036 | 1.014 | 1.058 | 1.009 | 1.004 | 1.015 | 1.139 | 1.055 | 1.230 | 1.039 | 0.999 | 1.081 |
|  |  | Lag0-7 | 1.041 | 1.017 | 1.066 | 1.009 | 1.004 | 1.015 | 1.214 | 1.128 | 1.305 | 1.065 | 1.025 | 1.106 |
|  | Cold |  |  |  |  |  |  |  |  |  |  |  |  |  |
|  |  | lag0 | 1.012 | 1.000 | 1.023 | 1.005 | 1.003 | 1.008 | 1.039 | 1.012 | 1.066 | 1.031 | 1.016 | 1.046 |
|  |  | lag1 | 1.022 | 1.009 | 1.035 | 1.001 | 0.998 | 1.005 | 1.043 | 1.012 | 1.075 | 1.023 | 1.005 | 1.040 |
|  |  | lag2 | 1.012 | 1.003 | 1.020 | 1.004 | 1.001 | 1.007 | 1.023 | 0.993 | 1.055 | 1.024 | 1.007 | 1.042 |
|  |  | lag3 | 1.010 | 1.004 | 1.015 | 1.003 | 1.000 | 1.006 | 1.024 | 0.993 | 1.055 | 1.013 | 0.996 | 1.031 |
|  |  | lag4 | 1.010 | 1.004 | 1.015 | 1.001 | 0.998 | 1.005 | 1.030 | 0.999 | 1.061 | 1.025 | 1.007 | 1.043 |
|  |  | Lag5 | 1.008 | 1.003 | 1.013 | 1.002 | 0.998 | 1.006 | 1.026 | 0.996 | 1.057 | 1.016 | 0.999 | 1.033 |
|  |  | Lag6 | 1.005 | 1.001 | 1.009 | 1.000 | 0.997 | 1.004 | 1.032 | 1.001 | 1.064 | 1.025 | 1.007 | 1.042 |
|  |  | Lag7 | 1.001 | 0.993 | 1.009 | 1.001 | 0.998 | 1.005 | 0.993 | 0.968 | 1.020 | 1.002 | 0.987 | 1.017 |
|  |  | Lag0-1 | 1.034 | 1.022 | 1.046 | 1.007 | 1.004 | 1.010 | 1.084 | 1.053 | 1.116 | 1.054 | 1.038 | 1.071 |
|  |  | Lag0-2 | 1.046 | 1.034 | 1.058 | 1.011 | 1.007 | 1.015 | 1.109 | 1.074 | 1.145 | 1.080 | 1.061 | 1.100 |
|  |  | Lag0-3 | 1.056 | 1.043 | 1.071 | 1.014 | 1.010 | 1.018 | 1.136 | 1.097 | 1.175 | 1.094 | 1.073 | 1.115 |
|  |  | Lag0-4 | 1.067 | 1.052 | 1.082 | 1.015 | 1.011 | 1.020 | 1.170 | 1.128 | 1.212 | 1.121 | 1.099 | 1.145 |
|  |  | Lag0-5 | 1.075 | 1.059 | 1.092 | 1.017 | 1.013 | 1.022 | 1.200 | 1.156 | 1.245 | 1.139 | 1.115 | 1.164 |
|  |  | Lag0-6 | 1.081 | 1.064 | 1.097 | 1.018 | 1.013 | 1.023 | 1.238 | 1.192 | 1.286 | 1.167 | 1.141 | 1.194 |
|  |  | Lag0-7 | 1.082 | 1.065 | 1.099 | 1.019 | 1.015 | 1.024 | 1.230 | 1.189 | 1.273 | 1.169 | 1.145 | 1.194 |
| pneumonia |  |  |  |  |  |  |  |  |  |  |  |  |  |  |
|  | Warm |  |  |  |  |  |  |  |  |  |  |  |  |  |
|  |  | lag0 | 1.003 | 0.990 | 1.016 | 0.999 | 0.996 | 1.003 | 1.002 | 0.953 | 1.053 | 0.990 | 0.967 | 1.014 |
|  |  | lag1 | 1.006 | 0.994 | 1.019 | 1.003 | 0.999 | 1.006 | 1.042 | 0.990 | 1.095 | 1.003 | 0.978 | 1.030 |
|  |  | lag2 | 1.008 | 0.999 | 1.018 | 1.002 | 0.998 | 1.005 | 1.045 | 0.993 | 1.099 | 1.016 | 0.989 | 1.043 |
|  |  | lag3 | 1.008 | 1.001 | 1.014 | 1.001 | 0.998 | 1.005 | 1.013 | 0.963 | 1.066 | 1.007 | 0.981 | 1.033 |
|  |  | lag4 | 1.006 | 1.000 | 1.013 | 1.001 | 0.997 | 1.005 | 0.973 | 0.925 | 1.023 | 0.996 | 0.970 | 1.022 |
|  |  | Lag5 | 1.006 | 1.000 | 1.012 | 1.001 | 0.998 | 1.005 | 1.030 | 0.980 | 1.082 | 1.015 | 0.989 | 1.042 |
|  |  | Lag6 | 1.006 | 1.001 | 1.011 | 1.003 | 0.999 | 1.007 | 1.056 | 1.006 | 1.108 | 1.019 | 0.993 | 1.046 |
|  |  | Lag7 | 1.007 | 0.997 | 1.017 | 1.000 | 0.997 | 1.003 | 1.066 | 1.018 | 1.116 | 1.024 | 1.001 | 1.048 |
|  |  | Lag0-1 | 1.009 | 0.994 | 1.024 | 1.002 | 0.998 | 1.005 | 1.043 | 0.978 | 1.113 | 0.994 | 0.965 | 1.023 |
|  |  | Lag0-2 | 1.017 | 1.001 | 1.034 | 1.004 | 0.999 | 1.008 | 1.090 | 1.013 | 1.172 | 1.010 | 0.977 | 1.043 |
|  |  | Lag0-3 | 1.025 | 1.007 | 1.044 | 1.005 | 1.000 | 1.009 | 1.105 | 1.022 | 1.193 | 1.017 | 0.980 | 1.054 |
|  |  | Lag0-4 | 1.032 | 1.012 | 1.052 | 1.006 | 1.000 | 1.011 | 1.075 | 0.990 | 1.166 | 1.012 | 0.974 | 1.052 |
|  |  | Lag0-5 | 1.038 | 1.016 | 1.060 | 1.007 | 1.001 | 1.012 | 1.107 | 1.018 | 1.204 | 1.027 | 0.986 | 1.070 |
|  |  | Lag0-6 | 1.044 | 1.020 | 1.069 | 1.010 | 1.004 | 1.016 | 1.169 | 1.073 | 1.274 | 1.047 | 1.003 | 1.093 |
|  |  | Lag0-7 | 1.052 | 1.025 | 1.079 | 1.010 | 1.004 | 1.016 | 1.246 | 1.148 | 1.353 | 1.072 | 1.028 | 1.118 |
|  | Cold |  |  |  |  |  |  |  |  |  |  |  |  |  |
|  |  | lag0 | 1.011 | 0.999 | 1.023 | 1.006 | 1.003 | 1.009 | 1.039 | 1.011 | 1.068 | 1.028 | 1.013 | 1.044 |
|  |  | lag1 | 1.023 | 1.009 | 1.037 | 1.001 | 0.998 | 1.005 | 1.047 | 1.014 | 1.081 | 1.027 | 1.008 | 1.045 |
|  |  | lag2 | 1.010 | 1.001 | 1.019 | 1.004 | 1.001 | 1.008 | 1.026 | 0.993 | 1.059 | 1.024 | 1.006 | 1.043 |
|  |  | lag3 | 1.009 | 1.003 | 1.015 | 1.003 | 1.000 | 1.007 | 1.014 | 0.982 | 1.047 | 1.011 | 0.993 | 1.030 |
|  |  | lag4 | 1.010 | 1.004 | 1.015 | 1.001 | 0.998 | 1.005 | 1.036 | 1.004 | 1.070 | 1.023 | 1.005 | 1.042 |
|  |  | Lag5 | 1.009 | 1.003 | 1.014 | 1.002 | 0.998 | 1.006 | 1.016 | 0.985 | 1.049 | 1.016 | 0.997 | 1.034 |
|  |  | Lag6 | 1.006 | 1.002 | 1.010 | 1.000 | 0.996 | 1.005 | 1.039 | 1.006 | 1.072 | 1.025 | 1.007 | 1.044 |
|  |  | Lag7 | 1.003 | 0.994 | 1.012 | 1.001 | 0.998 | 1.005 | 0.998 | 0.971 | 1.026 | 1.005 | 0.990 | 1.021 |
|  |  | Lag0-1 | 1.034 | 1.021 | 1.047 | 1.007 | 1.004 | 1.011 | 1.088 | 1.055 | 1.122 | 1.056 | 1.038 | 1.073 |
|  |  | Lag0-2 | 1.045 | 1.032 | 1.057 | 1.011 | 1.008 | 1.015 | 1.116 | 1.079 | 1.154 | 1.081 | 1.061 | 1.102 |
|  |  | Lag0-3 | 1.054 | 1.039 | 1.068 | 1.014 | 1.010 | 1.018 | 1.132 | 1.091 | 1.174 | 1.093 | 1.071 | 1.116 |
|  |  | Lag0-4 | 1.064 | 1.049 | 1.079 | 1.016 | 1.011 | 1.020 | 1.173 | 1.129 | 1.218 | 1.119 | 1.095 | 1.143 |
|  |  | Lag0-5 | 1.073 | 1.056 | 1.090 | 1.017 | 1.013 | 1.022 | 1.192 | 1.146 | 1.240 | 1.136 | 1.110 | 1.163 |
|  |  | Lag0-6 | 1.080 | 1.062 | 1.097 | 1.018 | 1.013 | 1.023 | 1.238 | 1.189 | 1.289 | 1.165 | 1.137 | 1.193 |
|  |  | Lag0-7 | 1.083 | 1.065 | 1.101 | 1.019 | 1.014 | 1.024 | 1.236 | 1.192 | 1.282 | 1.171 | 1.144 | 1.197 |
| bronchiolitis |  |  |  |  |  |  |  |  |  |  |  |  |  |  |
|  | Warm |  |  |  |  |  |  |  |  |  |  |  |  |  |
|  |  | lag0 | 1.002 | 0.983 | 1.021 | 1.000 | 0.994 | 1.005 | 1.025 | 0.955 | 1.100 | 0.982 | 0.946 | 1.018 |
|  |  | lag1 | 0.998 | 0.978 | 1.019 | 1.004 | 0.998 | 1.010 | 1.023 | 0.953 | 1.099 | 0.998 | 0.959 | 1.038 |
|  |  | lag2 | 0.988 | 0.973 | 1.003 | 0.994 | 0.988 | 1.000 | 1.012 | 0.941 | 1.087 | 1.016 | 0.976 | 1.057 |
|  |  | lag3 | 0.997 | 0.987 | 1.007 | 1.003 | 0.997 | 1.009 | 1.008 | 0.938 | 1.083 | 0.999 | 0.960 | 1.039 |
|  |  | lag4 | 1.006 | 0.997 | 1.016 | 1.003 | 0.997 | 1.008 | 0.980 | 0.912 | 1.053 | 0.998 | 0.959 | 1.038 |
|  |  | Lag5 | 1.009 | 0.999 | 1.018 | 1.001 | 0.995 | 1.006 | 0.969 | 0.903 | 1.039 | 0.987 | 0.949 | 1.027 |
|  |  | Lag6 | 1.005 | 0.997 | 1.013 | 1.002 | 0.996 | 1.008 | 1.072 | 1.002 | 1.147 | 1.031 | 0.991 | 1.073 |
|  |  | Lag7 | 1.000 | 0.985 | 1.014 | 1.000 | 0.995 | 1.006 | 1.063 | 0.996 | 1.134 | 1.027 | 0.992 | 1.063 |
|  |  | Lag0-1 | 1.000 | 0.978 | 1.024 | 1.004 | 0.998 | 1.010 | 1.049 | 0.959 | 1.148 | 0.979 | 0.937 | 1.024 |
|  |  | Lag0-2 | 0.988 | 0.963 | 1.014 | 0.998 | 0.990 | 1.005 | 1.061 | 0.958 | 1.176 | 0.995 | 0.946 | 1.046 |
|  |  | Lag0-3 | 0.985 | 0.956 | 1.015 | 1.000 | 0.993 | 1.008 | 1.070 | 0.959 | 1.192 | 0.994 | 0.941 | 1.050 |
|  |  | Lag0-4 | 0.992 | 0.961 | 1.023 | 1.003 | 0.994 | 1.011 | 1.048 | 0.934 | 1.176 | 0.991 | 0.935 | 1.051 |
|  |  | Lag0-5 | 1.000 | 0.967 | 1.034 | 1.004 | 0.995 | 1.013 | 1.015 | 0.901 | 1.143 | 0.979 | 0.920 | 1.042 |
|  |  | Lag0-6 | 1.005 | 0.971 | 1.041 | 1.006 | 0.997 | 1.015 | 1.088 | 0.965 | 1.227 | 1.010 | 0.946 | 1.078 |
|  |  | Lag0-7 | 1.005 | 0.966 | 1.045 | 1.006 | 0.997 | 1.016 | 1.157 | 1.033 | 1.295 | 1.037 | 0.973 | 1.105 |
|  | Cold |  |  |  |  |  |  |  |  |  |  |  |  |  |
|  |  | lag0 | 1.015 | 0.998 | 1.031 | 1.004 | 1.000 | 1.008 | 1.037 | 0.999 | 1.077 | 1.042 | 1.020 | 1.064 |
|  |  | lag1 | 1.019 | 1.001 | 1.038 | 1.002 | 0.997 | 1.007 | 1.031 | 0.987 | 1.077 | 1.005 | 0.979 | 1.031 |
|  |  | lag2 | 1.017 | 1.005 | 1.030 | 1.004 | 0.999 | 1.009 | 1.011 | 0.968 | 1.056 | 1.025 | 0.999 | 1.052 |
|  |  | lag3 | 1.015 | 1.007 | 1.023 | 1.004 | 0.999 | 1.008 | 1.066 | 1.020 | 1.114 | 1.021 | 0.995 | 1.048 |
|  |  | lag4 | 1.011 | 1.003 | 1.019 | 1.001 | 0.996 | 1.006 | 1.005 | 0.962 | 1.050 | 1.031 | 1.005 | 1.058 |
|  |  | Lag5 | 1.005 | 0.998 | 1.013 | 1.003 | 0.997 | 1.008 | 1.065 | 1.020 | 1.112 | 1.015 | 0.989 | 1.041 |
|  |  | Lag6 | 0.999 | 0.994 | 1.005 | 1.001 | 0.995 | 1.006 | 1.007 | 0.964 | 1.052 | 1.023 | 0.997 | 1.049 |
|  |  | Lag7 | 0.993 | 0.981 | 1.005 | 1.001 | 0.996 | 1.006 | 0.970 | 0.935 | 1.007 | 0.983 | 0.961 | 1.005 |
|  |  | Lag0-1 | 1.034 | 1.016 | 1.052 | 1.005 | 1.001 | 1.010 | 1.070 | 1.026 | 1.115 | 1.047 | 1.022 | 1.072 |
|  |  | Lag0-2 | 1.052 | 1.034 | 1.070 | 1.010 | 1.005 | 1.015 | 1.082 | 1.033 | 1.132 | 1.073 | 1.044 | 1.102 |
|  |  | Lag0-3 | 1.067 | 1.047 | 1.088 | 1.013 | 1.008 | 1.019 | 1.153 | 1.098 | 1.211 | 1.095 | 1.064 | 1.127 |
|  |  | Lag0-4 | 1.079 | 1.057 | 1.101 | 1.015 | 1.009 | 1.021 | 1.159 | 1.102 | 1.219 | 1.130 | 1.096 | 1.165 |
|  |  | Lag0-5 | 1.085 | 1.061 | 1.109 | 1.017 | 1.011 | 1.024 | 1.234 | 1.172 | 1.300 | 1.147 | 1.111 | 1.184 |
|  |  | Lag0-6 | 1.084 | 1.060 | 1.109 | 1.018 | 1.011 | 1.025 | 1.243 | 1.179 | 1.311 | 1.172 | 1.134 | 1.212 |
|  |  | Lag0-7 | 1.076 | 1.052 | 1.102 | 1.019 | 1.012 | 1.026 | 1.206 | 1.149 | 1.265 | 1.152 | 1.117 | 1.189 |

Table S6 Relative risk (95% CI) of ALRI, pneumonia and bronchiolitis hospitalizations associated with a 10 µg/m^3^ increase in air pollutant concentrations in single and two-pollutant models.

| Two-pollutant models | | ALRI | |  | Pneumonia | |  | Bronchiolitis ^a^ | |
| --- | --- | --- | --- | --- | --- | --- | --- | --- | --- |
|  |  | RR | 95%CI |  | RR | 95%CI |  | RR | 95%CI |
| PM_2.5_ | - | 1.089 | 1.075,1.103 |  | 1.094 | 1.079,1.109 |  | 1.067 | 1.048,1.086 |
|  | Adjusted for SO_2_ | 1.068 | 1.038,1.084 |  | 1.073 | 1.039,1.090 |  | 1.046 | 1.027,1.068 |
|  | Adjusted for NO_2_ | 1.053 | 1.038,1.067 |  | 1.054 | 1.039,1.070 |  | 1.047 | 1.027,1.068 |
| PM_10_ | - | 1.018 | 1.014,1.021 |  | 1.018 | 1.014,1.021 |  | 1.015 | 1.010,1.020 |
|  | Adjusted for SO_2_ | 1.013 | 1.007,1.014 |  | 1.014 | 1.007,1.017 |  | 1.010 | 1.006,1.016 |
|  | Adjusted for NO_2_ | 1.011 | 1.007,1.017 |  | 1.011 | 1.007,1.014 |  | 1.011 | 1.006,1.017 |
| SO_2_ | - | 1.186 | 1.154,1.219 |  | 1.188 | 1.154,1.224 |  | 1.185 | 1.133,1.239 |
|  | Adjusted for PM_2.5_ | 1.153 | 1.120,1.187 |  | 1.153 | 1.118,1.190 |  | 1.158 | 1.105,1.214 |
|  | Adjusted for PM_10_ | 1.174 | 1.142, 1.207 |  | 1.175 | 1.141,1.210 |  | 1.177 | 1.125,1.231 |
|  | Adjusted for NO_2_ | 1.139 | 1.142,1.174 |  | 1.139 | 1.141,1.176 |  | 1.154 | 1.125,1.210 |
| NO_2_ |  | 1.149 | 1.130,1.168 |  | 1.155 | 1.135,1.176 |  | 1.115 | 1.085,1.146 |
|  | Adjusted for PM_2.5_ | 1.135 | 1.116,1.155 |  | 1.140 | 1.119,1.161 |  | 1.104 | 1.074,1.136 |
|  | Adjusted for PM_10_ | 1.143 | 1.124,1,162 |  | 1.148 | 1.128,1.169 |  | 1.111 | 1.081,1.142 |
|  | Adjusted for SO_2_ | 1.139 | 1.124,1.160 |  | 1.146 | 1.128, 1.169 |  | 1.096 | 1.081,1.129 |

^a^ Noting: The maximum effect of air pollutants on each disease was lag0-7, except for SO_2_ and NO_2_ on bronchiolitis which was taken as lag0-6 for the maximum lag day.

Table S7 Relative risk (95% CI) in hospital admissions for ALRI, pneumonia and bronchiolitis associated with a 10μg/m^3^ increase in air pollutant concentrations in sensitivity analyses.

| Pollutants | Models | ALRI | Pneumonia | Bronchiolitis ^a^ |
| --- | --- | --- | --- | --- |
| PM_2.5_ |  |  |  |  |
|  | df=6 | 1.088(1.075,1.102) | 1.093(1.078,1.107) | 1.068(1.049,1.087) |
|  | df=7 | 1.089(1.076,1.103) | 1.094(1.079,1.108) | 1.068(1.049,1.087) |
|  | df=8 | 1.091(1.078,1.105) | 1.096(1.081,1.110) | 1.071(1.052,1.090) |
|  | df=9 | 1.092(1.079,1.106) | 1.096(1.082,1.111) | 1.073(1.054,1.092) |
|  | df=10 | 1.09(1.076,1.1040) | 1.095(1.08,1.110) | 1.067(1.047,1.087) |
| PM_10_ |  |  |  |  |
|  | df=6 | 1.018(1.015,1.022) | 1.018(1.015,1.022) | 1.016(1.011,1.021) |
|  | df=7 | 1.018(1.014,1.021) | 1.018(1.014,1.021) | 1.015(1.010,1.020) |
|  | df=8 | 1.018(1.014,1.021) | 1.018(1.014,1.022) | 1.015(1.010,1.021) |
|  | df=9 | 1.018(1.015,1.022) | 1.019(1.015,1.022) | 1.016(1.011,1.021) |
|  | df=10 | 1.017(1.013,1.020) | 1.017(1.013,1.021) | 1.014(1.009,1.019) |
| SO_2_ |  |  |  |  |
|  | df=6 | 1.172(1.140,1.204) | 1.173(1.139,1.208) | 1.177(1.125,1.23) |
|  | df=7 | 1.186(1.154,1.219) | 1.188(1.154,1.224) | 1.185(1.133,1.239) |
|  | df=8 | 1.177(1.145,1.209) | 1.179(1.145,1.215) | 1.174(1.122,1.227) |
|  | df=9 | 1.160(1.127,1.194) | 1.159(1.123,1.195) | 1.17(1.118,1.225) |
|  | df=10 | 1.175(1.142,1.208) | 1.180(1.145,1.216) | 1.163(1.111,1.216) |
| NO_2_ |  |  |  |  |
|  | df=6 | 1.142(1.123,1.162) | 1.148(1.128,1.169) | 1.112(1.082,1.142) |
|  | df=7 | 1.149(1.130,1.168) | 1.155(1.135,1.176) | 1.115(1.085,1.146) |
|  | df=8 | 1.143(1.125,1.162) | 1.150(1.130,1.170) | 1.109(1.079,1.139) |
|  | df=9 | 1.136(1.117,1.155) | 1.141(1.121,1.162) | 1.105(1.075,1.136) |
|  | df=10 | 1.144(1.126,1.163) | 1.152(1.132,1.172) | 1.106(1.076,1.137) |

^a^ Noting: The maximum effect of air pollutants on each disease was lag0-7, except for SO_2_ and NO_2_ on bronchiolitis which was taken as lag0-6 for the maximum lag day.
